# A Study of the Effect of 2 at.% Sn on the Microstructure and Isothermal Oxidation at 800 and 1200 °C of Nb-24Ti-18Si-Based Alloys with Al and/or Cr Additions

**DOI:** 10.3390/ma11101826

**Published:** 2018-09-25

**Authors:** Zhen Xu, Claire Utton, Panos Tsakiropoulos

**Affiliations:** Department of Materials Science and Engineering, Sir Robert Hadfield Building, The University of Sheffield, Mappin Street, Sheffield S1 3JD, UK; zhen_xu@outlook.com (Z.X.); c.utton@sheffield.ac.uk (C.U.)

**Keywords:** Niobium silicide-based alloys, solidification, oxidation, A15 intermetallics, silicides, solid solution, Laves phase, tin effect

## Abstract

Alloying with Al, Cr, Sn, and Ti significantly improves the oxidation of Nb silicide-based alloys at intermediate and high temperatures. There is no agreement about what the concentration of Sn in the alloys should be. It has been suggested that with Sn ≤ 3 at.% the oxidation is improved and formation of the brittle A15-Nb_3_Sn compound is suppressed. Definite improvements in oxidation behaviour have been observed with 5 at.% Sn or even higher concentrations, up to 8 at.% Sn. The research reported in this paper is about three model alloys with low Sn concentration and nominal compositions Nb-24Ti-18Si-5Cr-2Sn (ZX3), Nb-24Ti-18Si-5Al-2Sn (ZX5), and Nb-24Ti-18Si-5Al-5Cr-2Sn (ZX7) that were studied to understand the effect of the 2 at.% Sn addition on as-cast and heat-treated microstructures and isothermal oxidation in air at 800 and 1200 °C for 100 h. There was macrosegregation of Si and Ti in the alloys ZX3 and ZX5 and only of Si in the alloy ZX7. The Nb_ss_ was stable in all alloys. Tin and Ti exhibited opposite partitioning behaviour in the Nb_ss_. The βNb_5_Si_3_ was the primary phase in all three cast alloys and had partially transformed to αNb_5_Si_3_ in the alloy ZX3. Aluminium in synergy with Sn increased the sluggishness of the βNb_5_Si_3_ to αNb_5_Si_3_ transformation during solidification. After the heat treatment the transformation of βNb_5_Si_3_ to αNb_5_Si_3_ had been completed in all three alloys. Fine precipitates were observed inside some αNb_5_Si_3_ grains in the alloys ZX5 and ZX7. In the latter alloys the A15-Nb_3_X (X = Al, Si, and Sn) formed after the heat treatment, i.e., the synergy of Al and Sn promoted the stability of A15-Nb_3_X intermetallic in these Nb-silicide-based alloys even at this low Sn concentration. A Nb_ss_ + Nb_5_Si_3_ eutectic formed in all three alloys and there was evidence of anomalous eutectic in the parts of the alloys ZX3 and ZX7 that had solidified under high cooling rate and/or high melt undercooling. A very fine ternary Nb_ss_ + Nb_5_Si_3_ + NbCr_2_ eutectic was also observed in parts of the alloy ZX3 that had solidified under high cooling rate. At 800 °C none of the alloys suffered from catastrophic pest oxidation; ZX7 had a smaller oxidation rate constant. A thin Sn-rich layer formed continuously between the scale and Nb_ss_ in the alloys ZX3 and ZX5. At 1200 °C the scales formed on all three alloys spalled off, the alloys exhibited parabolic oxidation in the early stages followed by linear oxidation; the alloy ZX5 gave the smallest rate constant values. A thicker continuous Sn-rich zone formed between the scale and substrate in all three alloys. This Sn-rich zone was noticeably thicker near the corners of the specimen of the alloy ZX7 and continuous around the whole specimen. The Nb_3_Sn, Nb_5_Sn_2_Si, and NbSn_2_ compounds were observed in the Sn-rich zone. At both temperatures the scales formed on all three alloys consisted of Nb-rich and Nb and Si-rich oxides, and Ti-rich oxide also was formed in the scales of the alloys ZX3 and ZX7 at 1200 °C. The formation of a Sn-rich layer/zone did not prevent the contamination of the bulk of the specimens by oxygen, as both Nb_ss_ and Nb_5_Si_3_ were contaminated by oxygen, the former more severely than the latter.

## 1. Introduction

Materials with capabilities beyond those of Ni-based superalloys would allow future aero-engines to meet stringent environmental targets recommended by regulatory bodies, for example ACARE (Advisory Council for Aircraft Innovation and Research in Europe). Research towards the end of the last century demonstrated that Nb silicide-based alloys have the potential to replace Ni-based superalloys in aerofoil (blade) applications owing to their offering a balance of properties. The progress of this research was reviewed by Bewlay and Jackson [1]. The property goals that were identified by industry specified that new materials for high temperature applications in aero-engines must have acceptable oxidation. The long term oxidation goal is for a loss of <25 μm at 1315 °C [1]. The oxidation of developmental Nb-silicide-based alloys has steadily moved closer to the long term oxidation goal with different alloying strategies. The Nb-silicide-based alloys, as is the case for the Ni-based superalloys, will require an appropriate coating system to provide oxidation protection for long term service at 1200–1400 °C. Progress on Nb silicide-based alloys has been reviewed by one of the present authors [2]. For example, it was shown [2] that the actual compositions of some Nb-silicide-based alloys, of some Nb with no Si solid solutions observed in Nb-silicide-based alloys, and of some eutectics with Nb_ss_ and Nb_5_Si_3_ observed in Nb-silicide-based alloys satisfy the “standard definition” of the so-called “high-entropy alloys” (HEAs), “concentrated solid solution alloys” (CSSAs), “multi-principle element alloys” (MPEAs), and “complex concentrated alloys” (CCAs) (note that it is not suggested that all Nb-silicide-based alloys are HEAs).

The microstructures of Nb-silicide-based alloys contain the bcc Nb_ss_ and tetragonal Nb_5_Si_3_ but other phases such as tetragonal Nb_3_Si, C14-NbCr_2_ Laves, and A15-Nb_3_X (X = Al, Ge, Sn, and Si) intermetallics, can also be present depending on alloy composition. The alloying behaviour and properties of these phases were recently reviewed [3,4,5], where they were shown to be related to atomic size, electronegativity, and valence electron concentration. The development of Nb-silicide-based alloys has indicated that the addition of Al, Cr, Hf, and Ti significantly improves oxidation [6,7,8], which is further improved with the addition of Sn [9,10]. Considering oxidation, Al and Cr were added because of the importance of these elements in oxidation, Hf was added to act as a reactive element and to scavenge oxygen, and Ti was added to significantly reduce the diffusivity of oxygen in Nb [11].

Research has used different concentrations of Sn in Nb-silicide-based alloys. For example, some researchers have suggested that the Sn concentration should be about 3 at.% or less [6,7,8], while others have shown significant oxidation improvements with 2 at.% Sn [12] or 5 at.% Sn [9,12], or even higher concentrations, up to 8 at.% Sn [10]. The improvements in oxidation observed in alloys with Sn addition could be attributed to a number of reasons, for example, due to the fact that alloying additions decrease the diffusivity of oxygen in the Nb solid solution [6], or the observations (i) that Sn-rich phases form near the scale/diffusion zone interface [9,10], and (ii) that the volume fraction of Nb_ss_ decreases and A15-Nb_3_X intermetallic phases form in the cast microstructures and are stable after heat treatment when the concentration of Sn in the alloy exceeds 2 at.% [9,10,12].

It is not possible to identify the mechanism(s) responsible for the Sn effect on oxidation because all the research is on Nb-silicide-based alloys with solute additions of Al, Cr, Si, and Ti and other transition, refractory, simple metal, and metalloid additions plus Sn. For example, the two patents by Jackson et al. [7,8] are for multi-element alloys with solute additions that include Hf, one or more of the refractory metals Mo, Ta, and W, the simple metals, Sn and B, and metalloid element Ge. The alloys studied by Tsakiropoulos and co-workers include Hf, Mo, and Sn [9] or Fe, Hf, V, and Sn [12]. Knittel et al. [10] studied MASC type alloys with Hf and different Sn concentrations but with no additions of Ge and B (the MASC alloy nominal composition (at.%) is Nb-25Ti-16Si-8Hf-2Al-2Cr).

The motivation of the research presented in this paper was to understand how a low concentration of Sn in model Nb-24Ti-18Si silicide-based alloys with Al or Cr and Al and Cr addition(s) affects their microstructure and isothermal oxidation at 800 and 1200 °C. The nominal Sn concentration in the alloys was set at 2 at.%. We decided to avoid the addition of Hf in the alloys because we wished to investigate whether the bulk microstructures of the oxidised specimens would be contaminated by oxygen (Hf scavenges oxygen in Nb silicide-based alloys [6,9]). The structure of the paper is as follows. First, the microstructures of the cast and heat-treated alloys Nb-24Ti-18Si-5Cr-2Sn (ZX3), Nb-24Ti-18Si-5Al-2Sn (ZX5), and Nb-24Ti-18Si-5Al-5Cr-2Sn (ZX7) (nominal compositions, at.%) are discussed, followed by the results and discussion for their oxidation at 800 °C and 1200 °C.

## 2. Experimental

The alloys ZX3, ZX5, and ZX7 (see above for nominal compositions) were prepared using better than 99.99 wt.% purity elements and arc melting with a tungsten non consumable electrode and water cooled copper crucible in Argon atmosphere. Specimens for heat treatment were cut from the bulk of the 20 g buttons of each alloy, wrapped in Ta foil, and heat-treated under a constant flow of Ti gettered Argon at 1500 °C (ZX3 and ZX5) or 1450 °C (ZX7) for 100 h. The lower heat treatment temperature of the alloy ZX7 was selected because of liquation observed during heat treatment at 1500 °C. For the isothermal oxidation experiments at 800 °C and 1200 °C cubic (3 × 3 × 3 mm^3^) specimens were cut from the bulk of the buttons and ground to 1200 grit on each surface, and then placed in a NETZSCH STA 49 F3 Jupiter thermal analyser (NETZSCH GmbH, Selb, Germany) supported by the NETZSCH Proteus software. The instrument had a weight resolution of 0.1 µg over the entire weighing range (0–35,000 mg). A 3 °C/min heating rate from room temperature to 800 °C or 1200 °C was used.

For imaging and analysis, the alloy specimens were mounted in conductive Bakelite, ground to 2400 grit, and polished to a 0.25 µm finish. The oxidised alloy specimens were mounted in cold resin, ground to 2400 grit, and polished to a 0.25 µm finish. A Siemens 5000 X-ray diffractometer with monochromatic CuKa radiation was used to identify the phases in the cast and heat-treated alloys. The XRD specimens were ground to a 1200 grit finish and scanned with settings: 0.02° step and two theta (θ) from 20 to 100 degrees. The identification of phases used the JCPDS (Joint Committee of Powered Diffraction Standard) data.

The microstructures were observed using back scattered electron (BSE) imaging in an Inspect F SEM (ThermoFisher Scientific, Hillsboro, OR, USA). A Joel JSM 6400 SEM fitted with an Oxford instruments INCA (Oxon, UK) system was used for quantitative EDS analysis. At least 10 analyses were taken from each large area and phase. A Philips XF30 FEG SEM (Philips—ThermoFisher Scientific, Hillsboro, OR, USA) fitted with a Bruker Quantax analyser and ESPRIT software (Bruker AXS Ltd., Coventry, UK) was used to take X-ray maps. The software included data for N and O and assisted the identification for nitrides and oxides in the alloys. EPMA (Electron Probe Micro-Analysis) with Wave Dispersive Spectrometry (WDS) was used to identify the phases in the oxidised specimens. A Cameca SX100 instrument (Cameca, Gennevilliers, France) with spatial resolution of 1 µm was used to obtain the data. The instrument calibration was carried out by analysing reference materials with known compositions. The reference materials and their composition used in this work are listed in Table S1 of the Supplemental data. The maximum, minimum, and average values and the standard deviation are given in the Tables that present the analysis data.

## 3. Results

### 3.1. Cast Alloys

**Figure 1:**
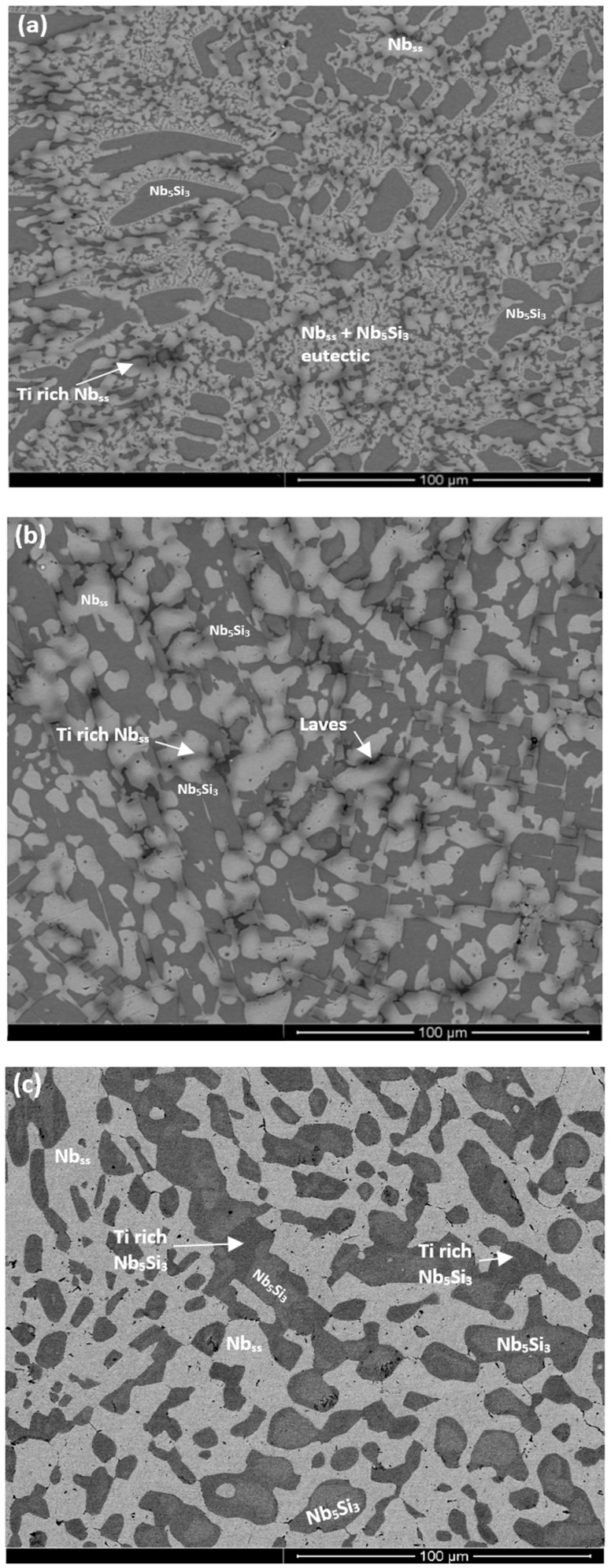
Back-scatter electron (BSE) images of the microstructure of the cast (**a**) bulk and (**b**) bottom of the button of the alloy ZX3 and (**c**) bulk of the heat-treated alloy.

**Figure 2:**
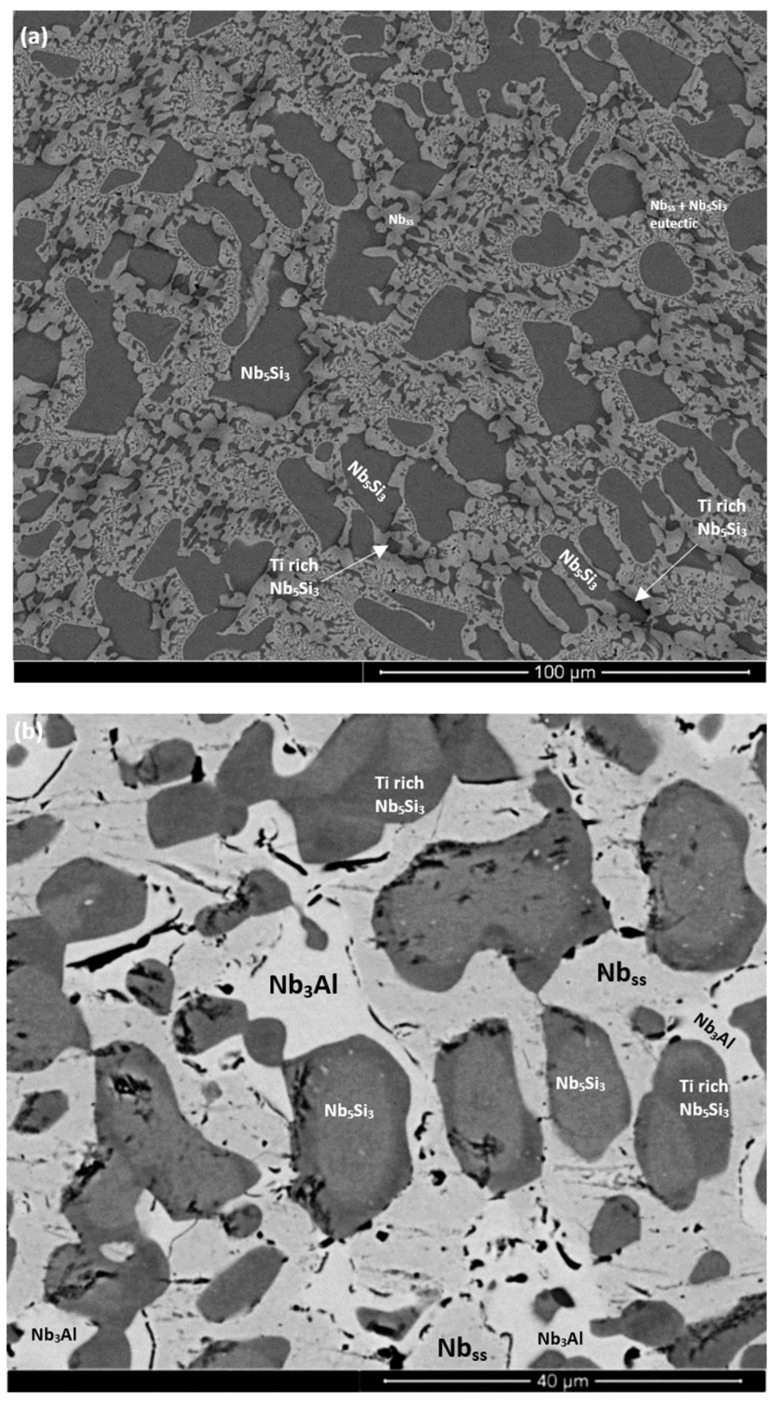
BSE images of the microstructure (**a**) of the bulk of the cast and (**b**) heat-treated alloy ZX5.

**Figure 3:**
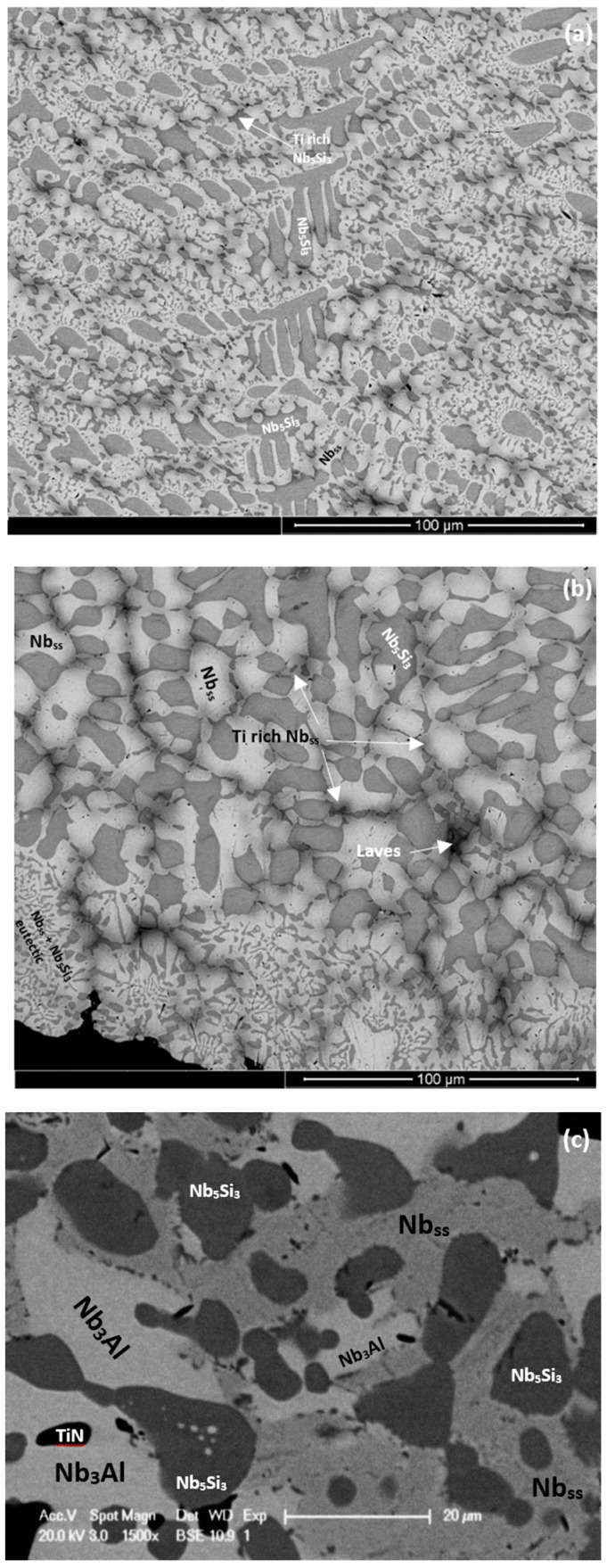
BSE images of the microstructure (**a**) of the bulk and (**b**) bottom of the cast and (**c**) heat-treated alloy ZX7.

The as-cast microstructures are shown in Figure 1a,b, Figure 2a, and Figure 3a,b, for the alloys ZX3, ZX5, and ZX7, respectively. The XRD data for the cast alloys is shown in the Supplemental data in Figure S1a,c,e, for alloys ZX3, ZX5, and ZX7, respectively. The actual compositions of the alloys and the phases present in their microstructures are summarised in Table 1. The actual compositions of the phases are given in the Supplemental data in Tables S2–S4 for the alloys ZX3, ZX5, and ZX7, respectively.

The alloys ZX3 and ZX5 were richer in Si compared with their nominal compositions. Control of the composition of Sn to 2 at.% proved very difficult when we prepared the alloy ZX7. After many attempts an alloy with the actual composition given in Table 1 was made, this was poorer in Si compared with the nominal composition. There was macrosegregation of Si and Ti in the alloys ZX3 and ZX5 and only of Si in the alloy ZX7. In ZX3 the Si and Ti concentrations varied respectively from 18.7 at.% to 22.3 at.% and 23.0 at.% to 26.8 at.%. In ZX5 the Si and Ti concentrations varied from 15.7 at.% to 21.3 at.% and 27.6 at.%, respectively. In ZX7 the Si concentration varied from 14.7 at.% to 17.7 at.%.

Nb_ss_ was formed in all three alloys and parts of the solid solution grains were rich in Ti (an example is shown in Figure S2 in the Supplemental data). βNb_5_Si_3_ was present in the cast microstructures of all alloys and had partially transformed to αNb_5_Si_3_ in the alloy ZX3. Nb_5_Si_3_ grains in ZX5 were rich in Ti. In the top and bulk of the buttons of the alloys ZX3 and ZX7, and in all parts of the button of the alloy ZX5, an Nb_ss_ + Nb_5_Si_3_ eutectic was observed. In the latter alloy the volume fraction of the Nb_ss_ + Nb_5_Si_3_ eutectic was higher in the bottom of the button. The Si + Al + Sn concentration of the Nb_ss_ + Nb_5_Si_3_ eutectic was about 19.8 at.% in the alloys ZX5 and ZX7, in agreement with a previous work [13].

The microstructure of the bottom area of the button of the alloy ZX3 was different compared with the near surface and bulk areas. The Nb_ss_ + Nb_5_Si_3_ eutectic was not observed, instead a co-continuous formation of Nb_ss_ and Nb_5_Si_3_ was found in this area, as shown in Figure 1b, similar to the anomalous eutectic that was observed in the bottom of the button of the alloy Nb-18Si-5Ge [14]. This microstructure gradually changed to that seen in the bulk and top of the ingot (Figure 1a). In the co-continuous microstructure observed in the bottom of the button of ZX3 there were dark contrast areas. These were confirmed by X-ray elemental maps to consist of a Cr-rich phase that was formed near the Ti-rich Nb_ss_ (Figure S2 in Supplemental data). These Cr-rich areas were present at a very small volume fraction and were too small to be analysed by EDS. They were attributed to the C14-NbCr_2_ Laves phase. The XRD did not confirm the presence of the latter owing to its low volume fraction. Furthermore, in the co-continuous microstructure observed in the bottom of the button of ZX3, a very fine eutectic structure was observed in which the Laves phase participated (Figure S3 in Supplemental data). This structure was attributed to the Nb_ss_ + Nb_5_Si_3_ + C14-NbCr_2_ Laves phase ternary eutectic that has been proposed by Bewlay et al. [15].

The EDS data (Table S2 in Supplemental data) and X-ray elemental maps for the alloy ZX3 (Figures S2 and S3 in Supplemental data) confirmed (i) that Ti partitioned to both the Nb_ss_ and Nb_5_Si_3_ where it substituted the Nb atoms, (ii) that the Laves phase was rich in Ti at the interfaces with the Nb_ss_ and Nb_5_Si_3_, (iii) that Cr and Sn partitioned to Nb_ss_ rather than Nb_5_Si_3_, and (iv) that the Cr concentration in the Nb_ss_ increased with the Ti concentration in the Nb_ss_ which would suggest an opposite partitioning behaviour for Ti and Sn in the Nb_ss_. The partitioning behaviour of Ti between the Nb_ss_ and Nb_5_Si_3_ and of Cr in the Nb_ss_ is in agreement with Zelenitsas and Tsakiropoulos [16].

In the bottom of the button of ZX7 the microstructure was different compared with the top and bulk. A zone approximately 50 μm thick that contained a Nb_ss_ + Nb_5_Si_3_ eutectic was formed in the areas where the melt had been in direct contact with the water cooled crucible (Figure 3b). Beyond this zone, a co-continuous structure of Nb_ss_ and Nb_5_Si_3_, observed in the alloy ZX3 (Figure 1b), was formed. In the co-continuous structure, a very low volume fraction of the Cr-rich phase was also observed (Figure S4 in Supplemental data and Figure 3b). These Cr-rich areas were attributed to the C14-NbCr_2_ Laves phase. The presence of a ternary eutectic, like the one in ZX3, was not confirmed.

### 3.2. Heat-Treated Alloys

The heat-treated microstructures are shown in Figure 1c, Figure 2b, and Figure 3c, for the alloys ZX3, ZX5, and ZX7, respectively. The XRD data for the heat-treated alloys is shown in the Supplemental data in the Figure S1b,d,f, for the alloys ZX3, ZX5, and ZX7, respectively. The actual compositions of the alloys and the phases present in their microstructures are summarised in Table 1. The actual compositions of the phases are given in the Supplemental data in Tables S2–S4, for the alloys ZX3, ZX5, and ZX7, respectively.

The Nb_ss_ was stable in all three alloys and was homogenised, meaning there were no solid solution grains with areas rich in Ti. After heat treatment the transformation of βNb_5_Si_3_ to αNb_5_Si_3_ was complete in all three alloys; Ti-rich Nb_5_Si_3_ was only observed in the alloy ZX3. There was no evidence of the prior eutectic microstructure. In the alloys ZX5 and ZX7 a new phase, namely A15-Nb3X (X = Al, Si, Sn), had formed. In the alloys ZX5 and ZX7 fine precipitates were observed inside some Nb_5_Si_3_ grains. These precipitates were very small in size and exhibited similar contrast to Nb_ss_ and the A15 intermetallic. In the alloy ZX7 the X-ray elemental maps (not shown) showed Cr-rich areas surrounding the Nb_ss_. These areas were attributed to the C14-NbCr_2_ Laves phase. Formation of Ti nitride was confirmed by X-ray elemental maps (not shown) in the microstructures of all three alloys, for example see Figure S1f in Supplemental data and Figure 2b.

The Si concentration in the Nb_ss_ was significantly reduced after heat treatment in all three alloys. The chemical composition of Nb_5_Si_3_ was essentially the same with the as-cast alloys ZX3 and ZX5, but in the alloy ZX7, the silicide became richer in Si and poorer in Al after heat treatment. The A15-Nb_3_X intermetallic phase was Al-rich and had Al + Si + Sn = 18.6 at.% in the alloys ZX5 and ZX7.

### 3.3. Oxidation at 800 °C

The weight gains after 100 h isothermal oxidation in air were 6, 6, and 3.5 mg/cm^2^ for the alloys ZX3, ZX5, and ZX7, respectively. The rate constants were calculated from the weight gain versus time data from the thermal analysis experiments (see Section 2) and are given in Table 2. None of the alloys suffered from catastrophic pest oxidation (Figure S5a,c,e in the Supplemental data). Parts of the thin scale that had formed on the specimen of ZX3 separated from the specimen leaving behind a clean surface and had formed the fine powder around the bottom of the specimen (Figure S5a in the Supplemental data). A few small parts of the scale formed on the specimen of ZX5 and were also seen around the specimen (Figure S5c in the Supplemental data). There was separation of the scale that formed on ZX7 (Figure S5e in the Supplemental data). ZX3 had followed linear oxidation kinetics throughout the experiment, and the oxidation of the alloys ZX5 and ZX7 exhibited parabolic oxidation kinetics, for approximately 50 and 42 h from the start of oxidation, respectively, followed by linear oxidation. The smaller rate constant values were exhibited by the alloy ZX7.

**Figure 4:**
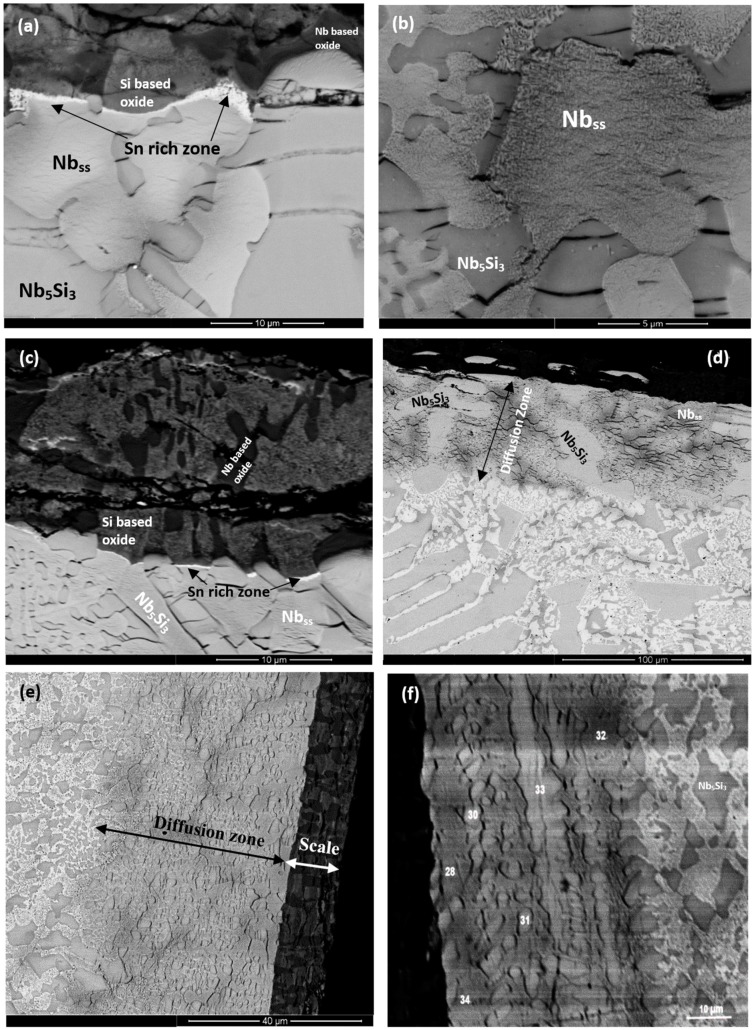
BSE images of cross-sections of the alloys ZX3 (**a**,**b**), ZX5 (**c**,**d**), and ZX7 (**e**,**f**) after isothermal oxidation for 100 h at 800 °C. The numbers in (**f**) link with the WDS analysis data in Table S7 in Supplemental data.

The oxides in the scales showed different contrasts under BSE imaging conditions. In the substrate beneath the scale an internal oxygen diffusion zone was present, as reported previously [6,17,18]. The data for the thickness of the scales, the oxides in the scales, the presence or absence of Sn-rich area/phase(s) at the scale/substrate interface (see below), the thickness of and the phases in the diffusion zone (see below), and the phases in the bulk of the oxidised specimens is summarised in Table 3. The WDS analysis data for the oxides in the scale and the phases in the diffusion zone bulk of the oxidised specimen is summarised in Tables S5–S7 in the Supplemental data. The Nb_ss_ and Nb_5_Si_3_ were contaminated by oxygen in the bulk of the oxidised specimens, the former more severely than the latter. The scales had cracks running parallel to the scale/substrate interface. In the diffusion zone the Nb_5_Si_3_ was cracked and the cracks run mostly parallel to the scale/substrate interface (for example see Figure 4a). The volume expansion of the oxidised Nb_ss_ led to the cracking of the Nb_5_Si_3_ (see Figure 4a,b) [6,17].

The microstructures of the oxidised alloys ZX3, ZX5, and ZX7 are shown in Figure 4a–f. In the case of ZX3, the Nb_ss_ in the diffusion zone was contaminated by oxygen and precipitates exhibiting dark contrast were formed inside the solid solution near the Nb_ss_/Nb_5_Si_3_ interfaces (Figure 4b), however in these areas the Nb_5_Si_3_ was not contaminated. In the bulk, both the Nb_ss_ and Nb_5_Si_3_ had been contaminated by oxygen, the former more severely (Table S5 in the Supplemental data). The Cr, Si, Sn, and Ti concentrations in Nb_5_Si_3_ were the same as in the cast alloy, which would suggest that Nb of the silicide had been consumed to form the oxide in the scale. The Nb and Si concentrations in the Nb_ss_ were lower compared with the cast alloy, which would suggest that the solid solution supplied Nb and Si for the oxides that formed in the scale.

In the case of the alloy ZX5, in the diffusion zone the Nb_5_Si_3_ and Nb_5_Si_3_ + Nb_ss_ eutectic were present and Nb_ss_ exhibited a similar contrast to Nb_5_Si_3_ (Figure 4c). Below the diffusion zone, the bulk microstructure was similar to the cast one. The EPMA data (Table S6 in the Supplemental data) would suggest that the light grey oxide was a Nb-rich oxide, and the dark grey oxide was a Nb and Si-rich oxide (Figure 4c). In the diffusion zone Nb_ss_ was more severely contaminated compared with Nb_5_Si_3_ (Table S6 in the Supplemental data) and the latter was cracked with the cracks running parallel to the scale/substrate interface. In the diffusion zone the oxygen concentration in the Nb_ss_ eutectic was approximately 34.8 at.%, which is 12 times that of Nb_5_Si_3_. Comparing the chemical compositions of the Nb_ss_ and Nb_5_Si_3_ in the cast alloy (Table S3 in the Supplemental data) shows that solute elements in the Nb_ss_ had been consumed for the formation of the scale and that Ti in the Nb_5_Si_3_ was also consumed. In the bulk of the oxidised specimen, the contamination of the microstructure was less severe, and in the eutectic, the Nb_ss_ and Nb_5_Si_3_ were easily distinguished (Figure 4d). In the bulk the oxygen concentration in the Nb_5_Si_3_ was the same as in the diffusion zone and the oxygen concentration in the Nb_ss_ had decreased to about 5.5 at.% (Table S6 in the Supplemental data).

In the case of the alloy ZX7, the light grey contrast oxide was Nb-rich, and the dark contrast oxide was Nb and Si-rich (Table S7 in the Supplemental data). In the diffusion zone the Nb_ss_ was heavily contaminated. The diffusion zone (Figure 4f) exhibited a “band” of “continuous” Nb_ss_ near the substrate/scale interface (analyses 28 and 34 in Table S7 in the Supplemental data) and a “band” of “continuous” Nb_5_Si_3_ approximately 15 to 20 µm below this interface (analysis 33), with “islands” of Nb_5_Si_3_ in between (analyses 30, 31). The analyses 28, 32, and 34 (Table S7 in the Supplemental data) from the heavily contaminated Nb_ss_ gave oxygen concentrations of approximately 33 at.%, which agrees well with that for the Nb_ss_ in the alloy ZX5 (approximately 35 at.%, Table S6 in the Supplemental data). The concentrations of Si and Sn in the contaminated Nb_ss_ were similar to those of the Nb_ss_ in the heat-treated alloy (Tables S4 and S7 in the Supplemental data). The lower Al, Cr, Nb, and Ti concentrations in the Nb_ss_ would suggest that these elements were consumed to form the oxides in the scale. The lower Si concentration in the contaminated Nb_ss_ compared with the bulk Nb_ss_ in the oxidised specimen would suggest that Si of the Nb_ss_ was also consumed for the formation of the scale. The analyses 30, 31, and 33 in Figure 4f were from the Nb_5_Si_3_ (Table S7 in the Supplemental data). The oxygen concentration in the Nb_5_Si_3_ was about 5 at.%, significantly lower than the Nb_ss_ in the same area. The concentrations of Al, Cr, Si, Sn, and Ti in the silicide were similar to those of Nb_5_Si_3_ in the cast alloy (Tables S4 and S7 in the Supplemental data).

In the alloys ZX3 and ZX5, a thin bright contrast layer was observed between the scale and the diffusion zone (for example, see Figure 4a). This layer formed continuously only between the oxide scale and Nb_ss_, in other words, it was not observed between the Nb_5_Si_3_ and scale. Owing to its thickness, it was not possible to quantify its composition. Based on the WDS analysis data for the oxidised alloys at 1200 °C, where this layer was thicker and could be analysed (see below), it is believed that this layer was Sn-rich and is identified as such in Figure 4a,c. In the case of the alloy ZX7 there was now clear evidence for the existence of such a Sn-rich layer at the scale/substrate interface.

### 3.4. Oxidation at 1200 °C

The weight gains after 100 h isothermal oxidation in air were 60, 30, and 35 mg/cm^2^, for the alloys ZX3, ZX5, and ZX7, respectively. The oxidised specimens are shown in Figure S5b,d,f in the Supplemental data and the rate constants are given in Table 2. The scales that were formed on all three alloys spalled off from all sides of the specimens. The oxidation of the alloys ZX3, ZX5, and ZX7 exhibited parabolic oxidation kinetics in the early stages of oxidation, followed by linear oxidation. The duration of parabolic oxidation increased from ZX3 to ZX5 to ZX7. The smaller rate constant values were exhibited by ZX5.

The data for the thickness of the scales, the oxides in the scales, the thickness of and the phases in the Sn-rich zone observed in the substrate at the scale/substrate interface, and the phases in the bulk of the oxidised specimens is summarised in Table 4. The WDS analysis data for the oxides in the scale and the phases in the Sn-rich zone and the bulk of the oxidised specimen is summarised in Tables S8–S11 in the Supplemental data. The Nb_ss_ and Nb_5_Si_3_ were contaminated by oxygen in the bulk of the oxidised specimens, the former more severely than the latter.

**Figure 5:**
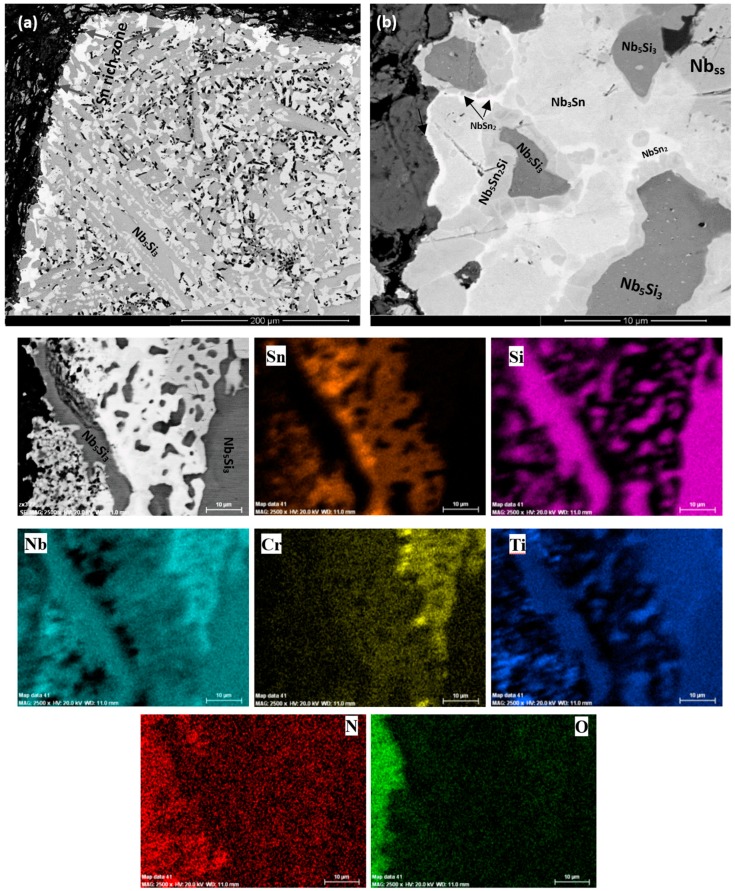
(**a**,**b**) BSE images of cross section, and X-ray elemental maps of the Sn rich zone of the oxidized alloy ZX3 at 1200 °C.

**Figure 6:**
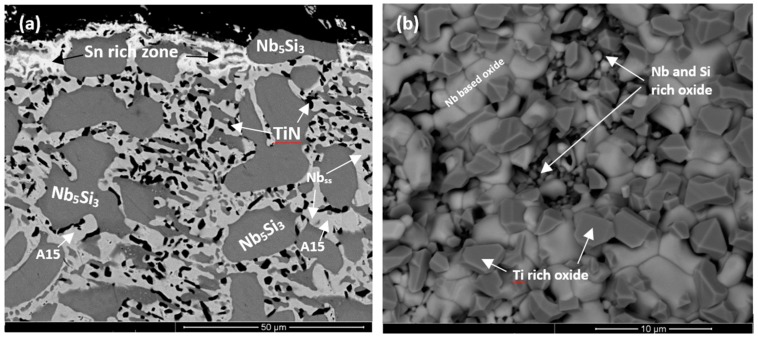

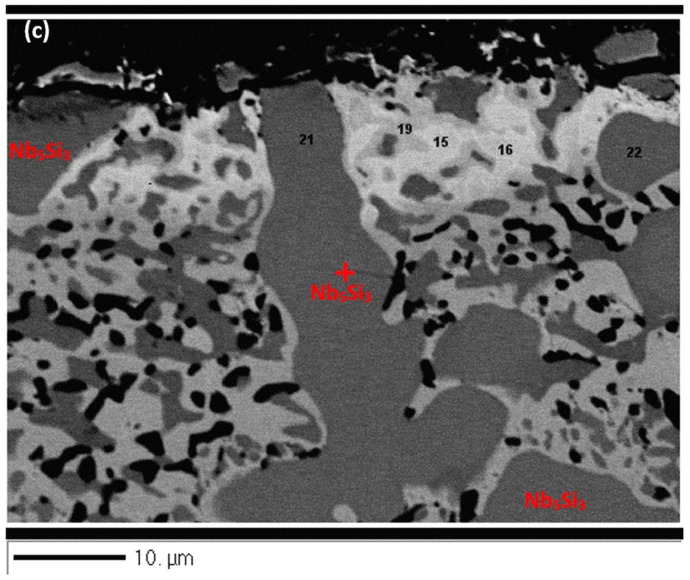
BSE images of cross-sections of the alloy ZX5 after oxidation for 100 h at 1200 °C. Sn-rich zone and bulk (**a**), scale (**b**), and details of the Sn-rich zone (**c**). The numbers in (**c**) link with the WDS analysis data in Table S9 in Supplemental data.

**Figure 7:**
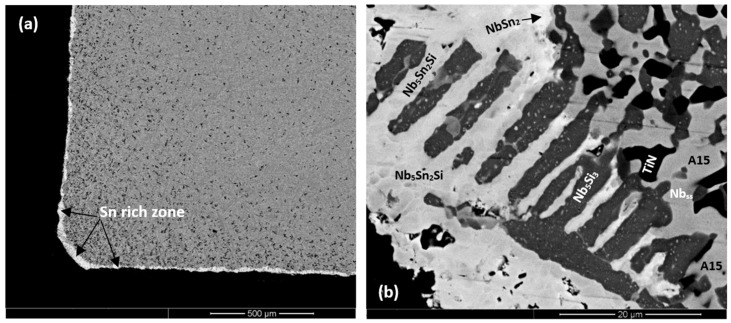

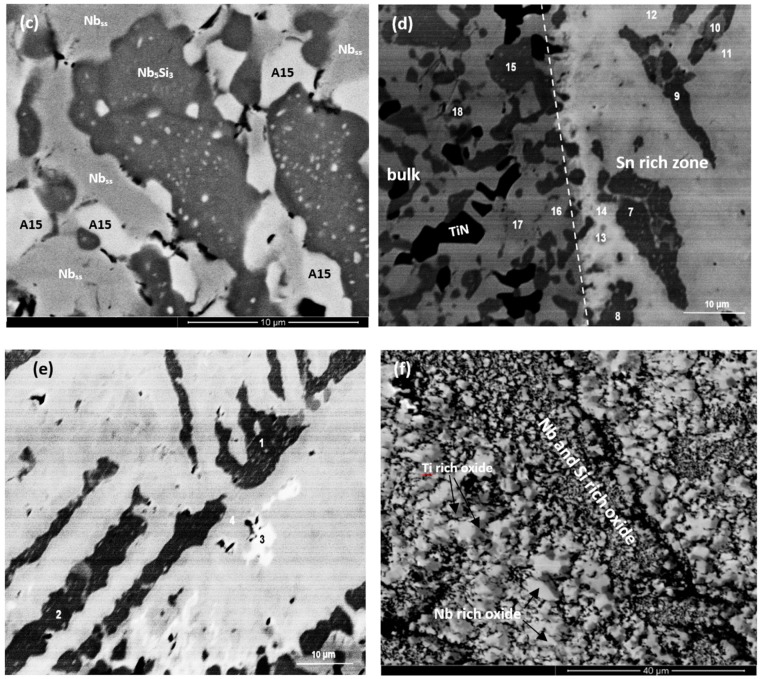
BSE images of the oxidised specimen of the alloy ZX7 at 1200 °C, (**a**) cross-section at low magnification showing the continuous Sn-rich zone, (**b**) Sn-rich zone, (**c**) bulk of specimen, (**d**) transition from the Sn-rich zone to the bulk of the specimen, (**e**) Sn-rich zone with points of analysis, (**f**) cross-section of scale. The numbers in (**e**,**d**) link with the WDS data in Tables S10 and S11 in Supplemental data.

Cross-sections of the oxidised specimens that clearly show a zone of white contrast are shown in Figure 5a, Figure 6a, and Figure 7a. In the case of ZX3, Figure 5 shows X-ray elemental maps for the Sn-rich zone. Compared with ZX3 at 800 °C (Figure 4a), the continuous Sn-rich zone formed at 1200 °C was thicker. Details of the Sn-rich zone are shown in Figure 5b where different Sn-rich phases are indicated. These phases were formed in between Nb_5_Si_3_ grains, and in the latter grains there was evidence of precipitation of a light contrast phase. Below the Sn-rich zone there was severe interstitial contamination of the alloy and Ti nitride (black contrast phase) was formed even in the bulk of the oxidised specimen. Ti nitrides were found both inside and on the grain boundaries of the Nb_ss_. In the scale Nb-rich, Ti-rich, and Nb and Si-rich oxides were observed (Table S8 in the Supplemental data). The EPMA analysis data confirmed that the Nb_3_Sn, Nb_5_Sn_2_Si, and NbSn_2_ compounds were present in the Sn-rich zone (Table S8 in the Supplemental data). In the bulk both the Nb_ss_ and Nb_5_Si_3_ were contaminated by oxygen, the former more severely (Table S8 in the Supplemental data). Compared with the data for 800 °C, the composition of the Nb_5_Si_3_ was essentially the same, but the oxygen concentration in the Nb_ss_ was higher (Tables S5 and S8 in the Supplemental data).

Figure 6a shows a cross-section of the alloy ZX5. Details of the Sn-rich zone are shown in the Figure 6c. The presence of Ti nitride and A15-Nb_3_X and Nb_ss_ phases was confirmed by X-ray elemental maps (not shown). The Nb_ss_ and A15-Nb_3_X phases exhibited similar contrast under BSE imaging conditions and were differentiated from each other using X-ray elemental maps and quantitative analysis. The scale (Figure 6b) was composed of different oxides (Table S9 in the Supplemental data). Porosity was observed close to the top surface of the scale but the microstructure was compact in the bulk of the scale. The chemical composition of the oxides was close to that of oxides formed at 800 °C (Tables S6 and S9 in the Supplemental data). The Sn-rich zone is shown in Figure 6c. EPMA analyses confirmed that the Nb_5_Si_3_ (analyses 21, 22) was not heavily contaminated by oxygen and that the Sn-rich intermetallic phases were Nb_3_Sn (analyses 15, 16) and probably Nb_5_Sn_2_Si (analysis 19) (Table S9 in the Supplemental data). In the bulk, the oxygen concentration in the Nb_ss_ was approximately 8 at.%, compared with 5 at.% at 800 °C. The oxygen concentration in the Nb_5_Si_3_ had increased slightly, from approximately 3 at.% at 800 °C to approximately 4 at.% at 1200 °C.

The Sn-rich zone formed in the alloy ZX7 (Figure 7a and Figure S6 in the Supplemental data) was noticeably thicker near the corners, was formed along the edges of the specimens, similar to the Sn-rich zones in the alloys ZX3 and ZX5 but in the case of the alloy ZX7 the zone was continuous around the whole specimen. The transition between the Sn-rich zone and the bulk is shown in Figure 7d and the microstructure of the scale is shown in Figure 7f. The oxides were Nb-rich, Ti-rich, and Nb and Si-rich (Table S10 in the Supplemental data). Figure 7b shows different contrasts in the Sn-rich zone, similar to the alloys ZX3 (Figure 5) and ZX5, which indicated high and low Sn concentrations, respectively. There were also changes in the bulk of Nb_5_Si_3_ grains in the Sn-rich zone (Figure 7b) and in the bulk of the specimen (Figure 7c). X-ray elemental maps (not shown) confirmed that the phase(s) inside the Nb_5_Si_3_ were Sn-rich. The identity of phases in the Sn-rich zone is shown in Figure 7b. Considering Figure 7e, analyses 1 and 2 correspond to the Nb_5_Si_3_. Analysis 3 of the very bright contrast phase was attributed to the Sn-rich NbSn_2_ compound. Analysis 4 was attributed to the Nb_5_Sn_2_Si compound (Table S10 in the Supplemental data).

The transition from the Sn-rich zone to the bulk of ZX7 is shown in Figure 7d. The EPMA data is given in Table S11 in the Supplemental data. Analyses 7, 8, 9, and 10 corresponded to Nb_5_Si_3_ in the Sn-rich zone. The Nb_5_Si_3_ had Si + Al + Sn in the range 35.1 to 36.7 at.%. The Sn-rich Nb_5_Si_3_ (analysis 10) is consistent with the formation of Sn-rich areas inside the Nb_5_Si_3_, as seen in Figure 7b and Figure S6 in the Supplemental data. Analyses 7 and 9 provided further evidence for the formation of Sn-rich areas inside the Nb_5_Si_3_. There was some kind of separation of Nb_5_Si_3_ in subgrains with Sn-rich subgrain “boundary areas”, see Figure S6 in the Supplemental data. The analyses 11 and 12 would suggest the presence of oxidised Nb_5_Sn_2_Si. Analyses 13 and 14 were taken closer to the interface (dashed line in Figure 7d to indicate the transition from the Sn-rich zone to the bulk) and are richer in Sn with Sn/(Si + Al), with a ratio close to 2:1; they could also be oxidised Nb_5_Sn_2_Si since Nb_5_Sn_2_Si and Nb_5_Sn_2_Al have the same structure. Below the Sn-rich zone, the Nb_5_Si_3_ (analysis 15) and Al-rich A15-Nb_3_X phase (analyses 16 and 17) were studied. The Al concentration in the latter phase was approximately 10 at.% and the Al + Si + Sn was 18 at.%. The Laves phase was also present in this area (analysis 18, see also Figure S6 in the Supplemental data) and contained 49.8 at.% Cr, with Cr + Al + Si + Sn = 57.9 at.%. In the bulk microstructure, the Nb_5_Si_3_, A15-Nb_3_X and Nb_ss_ phases were present as well as the C14-NbCr_2_ Laves phase. The X-ray maps of the Sn-rich zone (Figure S6 in the Supplemental data) and bulk of the alloy (not shown) confirmed the presence of C14-NbCr_2_ Laves phase.

## 4. Discussion

### 4.1. Macrosegregation of Si

The macrosegregation of an element i in Nb-silicide-based alloys has been defined as MACi = C_max_^i^ − C_min_^i^, i.e., as the difference between the maximum and minimum concentrations of the element in the alloy [19]. The macrosegregation of elements in cast Nb-silicide-based alloys was discussed previously [20], where it was linked with the partitioning of solutes between phases in the microstructure of Nb silicide-based alloys, namely the Nb_ss_, Nb_5_Si_3_, NbCr_2_ Laves, and the Nb_ss_ + Nb_5_Si_3_ eutectic.

The macrosegregation of Si in Nb-silicide-based alloys with different microstructures (meaning alloys with different phases and volume fractions of phase(s)) was studied previously [20] using different material parameters. The ranking of Nb-silicide-based alloys in terms of increasing Si macrosegregation (MACSi) indicated that the latter tended to increase when the parameters ΔH_m_/T_m_ (“alloy entropy of fusion”), T_m_^sp^ (melting temperature of sp electronic configuration elements), and (ΔH_m_/T_m_)(ΔH_m_^sd^/ΔH_m_^sp^)^−1^ increased, and the parameters ΔH_m_^sd^/ΔH_m_^sp^, T_m_^sd^/T_m_^sp^, ΔH_m_ (“alloy enthalpy of melting”), T_m_ (alloy melting temperature), and T_m_^sd^ (melting temperature of the sd electronic configuration elements) decreased.

How does Sn affect the macrosegregation of Si in Nb-24Ti-18Si-based alloys? To answer this question, the aforementioned parameters for the previously studied “reference” alloys without Sn, namely the alloys KZ4, KZ7, and KZ5 [16], and the parameters of the alloys of this study are compared in the Table 5, where the nominal compositions of KZ4, KZ5, and KZ7 are given. In Table 5, the values of the parameters that follow the trends mentioned above are shown in bold. The trends are followed with the exception of the parameter ΔH_m_ for the alloys ZX3, ZX5, and ZX7 and the parameters T_m_^sp^ and T_m_^sd^/T_m_^sp^ for the latter alloy.

The data in Table 5 shows that the addition of 2 at.% Sn increased the macrosegregation of Si, and that the latter is linked with increase of the parameters ∆H_m_/T_m_ and (ΔH_m_/T_m_)(ΔH_m_^sd^/ΔH_m_^sp^)^−1^ and decrease of the parameters T_m_, ΔH_m_^sd^/ΔH_m_^sp^, and T_m_^sd^. The differences with the aforementioned trends for ∆H_m_, T_m_^sp^, and T_m_^sd^/T_m_^sp^, seen in the Table 5, are attributed to differences with the compositions of the “reference” alloys. For example, the alloys ZX3 and ZX5 were significantly richer in Si compared with the alloys KZ4 and KZ7, and the alloys ZX4 and ZX7 were poorer in Cr compared with the alloys KZ4 and KZ5 [16].

**Figure 8:**
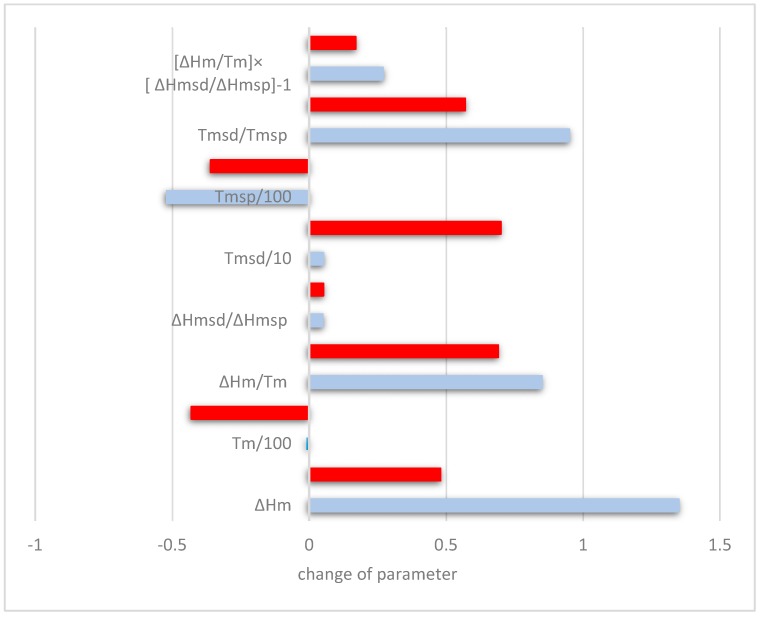
Effect of Al (red) and Cr (blue) on the reduction of macrosegregation of Si. Positive change of parameter value indicates that the addition of Cr or Al increased the specific parameter while reducing MACSi (see text).

How did the synergies of Al and/or Cr with Sn affect the macrosegregation of Si in Nb-24Ti-18Si-based alloys? The data for MACSi for the “reference” alloys KZ4, KZ5, and KZ7 showed that simultaneous addition of Al and Cr reduced the macrosegregation of Si and that Al had a stronger effect on MACSi than Cr. The addition of Sn did not cancel out the effects of Al and Cr when these elements were in synergy with 2 at.% Sn on their own or simultaneously, but the synergy of Al and Sn had the strongest effect on MACSi (Cr and Sn and Al + Cr + Sn increased MACSi by 1.7 at.%, and Al and Sn increased MACSi by 3.2 at.%). The data for the alloys ZX3, ZX5, and ZX7 can be used to find out how Al or Cr addition affected the aforementioned parameters; this is shown in Figure 8. For example, when Cr was added to ZX7, a comparison of the data for the alloys ZX5 and ZX7 shows that the macrosegregation of Si was reduced. This reduction of MACSi was accompanied by increase (positive changes) of the parameters ΔH_m_, ΔH_m_/T_m_, ΔH_m_^sd^/ΔH_m_^sp^, T_m_^sd^, T_m_^sd^/T_m_^sp^, and (ΔH_m_/T_m_)(ΔH_m_^sd^/ΔH_m_^sp^])^−1^. Note that in order to compare the parameters in the Figure 8, the data for the changes of T_m_ and T_m_^sp^ is divided by 100 and the data for the change in T_m_^sd^ is divided by 10.

### 4.2. Microstructures

The phases present in the alloys of the as-cast and heat-treated conditions are summarised in Table 1. The compositions of the Nb_ss_, Nb_5_Si_3_ and A15-Nb_3_X intermetallic phases, and Nb_ss_ + Nb_5_Si_3_ eutectics are compared in Table 6.

#### 4.2.1. Cast Microstructures

The microstructure in the bottom of the button of alloy ZX3 was different compared with the bulk and top. The Nb_ss_ + Nb_5_Si_3_ eutectic was not observed (Figure 1b), instead, there was co-continuous Nb_ss_ and Nb_5_Si_3_ with a very small volume fraction of C14-NbCr_2_ Laves phase in between Nb_ss_ dendrites (Figure S2 in Supplemental data) as well as a very small volume fraction of a ternary eutectic (Figure S3 in Supplemental data). Compared with the “reference” alloy KZ4 [16], there was agreement regarding the location of the formation of the C14-NbCr_2_ Laves phase was observed in the microstructure (i.e., in the last to solidify Cr-rich melt in between Nb_ss_ dendrites), but there was disagreement regarding where the C14-NbCr_2_ Laves phase was observed. Indeed, in the alloy KZ4 the C14-NbCr_2_ Laves phase was observed in all parts of the 600 g button, but in alloy ZX3 was only observed in the bottom. The former alloy was richer in Cr than the latter in all parts of the larger button (7–7.5 at.% vs. 4–4.9 at.%), and in ZX3 the melt near the bottom was richer in Cr than the top (4.9 vs. 4 at.%); but still poorer in Cr compared with the alloy KZ4. This would suggest that in ZX3 the formation of the C14-NbCr_2_ Laves phase depended on the synergy of Sn with Cr and Ti, and solidification conditions.

The composition in the bottom of the button of alloy ZX3 was Nb-26Ti-20.4Si-4.9Cr-2.2Sn with Si + Sn = 22.6 at.%, i.e., close to the composition of the metastable Nb_ss_ + Nb_5_Si_3_ eutectic [13]. The melt that was in contact with the water cooled copper crucible solidified as an anomalous eutectic (Figure 1b) and, further from the crucible wall, there was transition to anomalous + normal, then to normal eutectic, and to the microstructure seen in the bulk of the ingot (Figure 1a). Transitions between anomalous and normal eutectics have been reported to occur in the unconstrained (i.e., free) solidification of bulk undercooled binary eutectic alloys where facetted—non-faceted (f-nf) and non-faceted—non-faceted (nf-nf) eutectics are formed. Furthermore, a refinement of anomalous eutectic microstructure and increase of its volume fraction have been reported with increasing undercooling for f-nf eutectics and nf-nf eutectics [21,22]. At least seven mechanisms have been suggested for anomalous eutectic formation. Kuribayashi and co-workers have attributed the aforementioned transition to the decoupled growth of the two phases owing to their different entropies of fusion [23,24] where the non-faceted (low entropy of fusion) phase outgrows the facetted (high-entropy of fusion) phase after eutectic growth starts.

Close to the crucible wall, the growth of Nb_ss_ was easier than that of the βNb_5_Si_3_ owing to the higher entropy of fusion of the latter (ΔS_f_^Nb5Si3^ ≈ 14.55 J/mol·K) compared with the former (ΔS_f_^Nb^ ≈ 9.45 J/mol·K). As the growth of both Nb_ss_ and βNb_5_Si_3_ progressed under decreasing melt undercooling (i.e., as solidification progressed away from the crucible wall towards the bulk of the button) there was rejection of Cr, Sn, Ti, and of Si to the melt from the βNb_5_Si_3_ and the Nb_ss_, respectively, and in the Cr-rich parts of the melt the C14-NbCr_2_ Laves phase formed, while in the areas of the melt where the composition reached that of the ternary Nb_ss_ + NbCr_2_ + βNb_5_Si_3_ eutectic, the latter formed. A ternary eutectic in Nb-Si-Cr alloys, involving the Nb_ss_ and Laves phases, has been suggested by Bewlay et al. [15]. This eutectic in Nb-Si-Cr alloys has not been confirmed by other researchers and its formation is believed to depend on solidification (cooling) conditions. Ternary eutectics were observed in the interdendritic areas that were possibly composed of the Nb_ss_ + Nb_5_Si_3_ + C14-NbCr_2_ Laves phases, as suggested by Yang et al. for the solidification of the Nb-23Ti-15Si-10Cr alloy [25]. Thus, it is suggested that in the bottom of the button of the alloy ZX3 the solidification path was L → L + Nb_ss_ + βNb_5_Si_3_ → L + Nb_ss_ + βNb_5_Si_3_ + NbCr_2_ (interdendritic) → Nb_ss_ + βNb_5_Si_3_ + NbCr_2_ + (Nb_ss_ + βNb_5_Si_3_ + NbCr_2_)_ternary eutectic_ compared with the L → L + βNb_5_Si_3_ → βNb_5_Si_3_ + (Nb_ss_ + βNb_5_Si_3_)_eutectic_ → βNb_5_Si_3_ + αNb_5_Si_3_ + (Nb_ss_ + βNb_5_Si_3_)_eutectic_ solidification path in the bulk of the button.

The microstructure in the bottom of the button of the alloy ZX7 showed a zone approximately 50 μm thick where the lamellar Nb_ss_ + Nb_5_Si_3_ eutectic was formed next to the crucible wall; above this zone an anomalous eutectic was formed (Figure 3b) and then the Nb_5_Si_3_ + (Nb_ss_ + Nb_5_Si_3_)_eutectic_ microstructure in the bulk and top of the button (Figure 3a). Anomalous eutectic was not observed in the cast alloy ZX5 (Al present, but no Cr), where the volume fraction of the lamellar Nb_ss_ + Nb_5_Si_3_ eutectic was higher in the bottom areas of the button compared with the bulk and top. Furthermore, as we discussed above, an anomalous eutectic was observed in the bottom of the cast alloy ZX3 (Cr present, but no Al), where the C14-NbCr_2_ Laves phase was also formed, as was the case in the anomalous eutectic in the bottom of the button of the alloy ZX7 (Figure 3b). Considering the data for the alloys ZX5 and ZX3, it was suggested that near the water cooled crucible wall the aforementioned zone was formed from an Al-rich melt of eutectic composition solidifying as a lamellar eutectic, while the Cr-rich eutectic melt solidified as anomalous eutectic.

The microstructure in the bottom of the button of the alloy ZX7 and the aforementioned transition in eutectic microstructures with increased melt undercooling would also suggest that the microstructure in the 50 μm thick zone (Figure 3b) was formed from melts that solidified under different melt undercooling near the crucible wall. Assuming that the temperature of the melt in contact with the crucible wall was T_melt_^crucible^ and that the eutectic temperatures T_eutectic_ for Cr or Al-rich melts were different, the undercooling (∆T = T_eutectic_ − T_melt_^crucible^) was ∆T_Al-rich melt_ < ∆T_Cr-rich melt_. Assuming that nucleation of solidification occurred first at the lower undercooling, as the Nb_ss_ + Nb_5_Si_3_ lamellar eutectic formed from the Al-rich melt, the surrounding melt became richer in Si, (see Table S4 in the Supplemental data and note that the Ti, Al, Si, and Sn concentrations of the eutectics in the alloys ZX5 and ZX7 are essentially the same, Table S3 in the Supplemental data) and from this richer-in-Si melt formed the anomalous eutectic at higher melt undercooling (note that the Ti and Sn concentrations of the eutectic in the alloy ZX3 are essentially the same as in the eutectics in the alloys ZX5 and ZX7 but the eutectic in ZX3 was richer in Si, Table S2 in the Supplemental data).

It is reasonable to assume that the growth velocity V_S/L_ was “constant” during the solidification of the 50 μm zone (growth velocity “imposed” by the conditions near the crucible wall). According to the model of Tiller et al. [26] for the solute concentration (C_L_*) at the S/L interface during the initial transient solidification, the C_L_* is proportional to the solute concentration in the melt C_o._ and the constitutional undercooling ∆T_CS_ during the initial transient is given by the equation [27]:

∆T_CS_ = m_L_[C_o._/k_o_](1 − k_o_)[1 − exp(−k_o_V_S/L_^2^t/D_L_)][1 − exp(−V_S/L_x/D_L_)] − Gx (1)

where t is time, x is distance from S/L interface, D_L_ is diffusion coefficient in the melt, k_o_ is the partition coefficient, G is the temperature gradient, and m_L_ is the liquidus slope. In other words, as the melt became rich in Si ahead of the lamellar eutectic (i.e., C_o._ increased in the above equation) the ∆T_CS_ increased and thus the anomalous eutectic formed. The latter grew over some distance in excess of 50 μm (Figure 3b) and eventually the transition to the normal eutectic in the bulk of the ingot occurred, for the same reasons as discussed above for the alloy ZX3.

Therefore, the microstructures in the bottom areas of the buttons of the alloys ZX3, ZX5, and ZX7 would suggest (i) that Cr had a strong effect (i.e., promoted or stabilised) on the anomalous to lamellar eutectic transition (that occurred with decreasing melt undercooling), (ii) that the addition of Al did not stabilise the anomalous eutectic, and (iii) that it is possible to have a co-continuous Nb_ss_ and Nb_5_Si_3_ microstructure formed from deeply undercooled Cr containing eutectic melts, even in the presence of Al. The additions of Al and Cr, individually or simultaneously, to the alloys did not destabilise Nb_ss_ in all of the alloys and did not suppress the partitioning of Ti in the Nb_ss_ during solidification. Indeed, Ti-rich areas in the Nb_ss_ (i.e, partitioning of Ti in Nb_ss_) were observed in all cast alloys. Furthermore, the additions of Al and Cr individually or simultaneously in the alloys also did not destabilise the Nb_ss_ + βNb_5_Si_3_ eutectic in all the alloys.

Only Cr on its own promoted the βNb_5_Si_3_ to αNb_5_Si_3_ transformation during solidification in the alloy ZX3 and Al destabilised this effect of Cr in the alloy ZX7; in the “reference” alloy KZ7 [16] only the βNb_5_Si_3_ formed in the cast microstructure, the same was the case for the cast alloy ZX5. Zelenitsas and Tsakiropoulos [16] proposed that Al stabilises βNb_5_Si_3_ during solidification. This is supported by the data for ZX5 (Figure S1c in the Supplemental data), in other words, Al in synergy with Sn increased the sluggishness of the βNb_5_Si3 → αNb_5_Si_3_ transformation during solidification.

#### 4.2.2. Heat-treated Microstructures

In all alloys αNb_5_Si_3_ was the stable silicide after the heat treatment. In the alloys with Al, after heat treatment the βNb_5_Si_3_ → αNb_5_Si_3_ transformation had been completed, and in some of the silicide grains, a second phase had precipitated. The fine precipitates in αNb_5_Si_3_ grains exhibited contrast similar to that of the Nb_ss_ and A15-Nb_3_X. The limited evidence from this research was not strong enough to categorically state what the precipitates are. The formation of fine precipitates in αNb_5_Si_3_ is consistent with the data for the “reference” alloys KZ7 and KZ5 [16], where it was suggested (based on the contrast of the fine precipitates) that the precipitates were Nb_ss_ formed via the transformation βNb_5_Si_3_ → Nb_ss_ + αNb_5_Si_3_. Precipitation of Nb_ss_ in αNb_5_Si_3_ was further discussed recently [28].

Aluminium, like Si and Sn, can form A15-Nb_3_X (X = Al, Si, Sn) compounds and Cr can form A15-Cr_3_Si. The A15-Nb_3_X intermetallic phase that formed after the heat treatment was rich in Al and poor in Cr and was observed only when Al was present in the alloys. In the alloys ZX5 and ZX7, where EDS analysis of this phase was possible, the Si + Sn and Si + Sn + Al sums and the Si/Sn ratio were ≈10 at.%, ≈18.7 at.%, and 0.9, respectively, see Table 6. The data for the A15 phase in the alloys ZX5 and ZX7 is in agreement with a past work [5]. The formation of the A15-Nb_3_X compound would suggest that the synergy of Al and Sn can promote the stability of A15-Nb_3_X even at low Sn concentrations in Nb silicide-based alloys. The addition of Al did not destabilise the C14-NbCr_2_ Laves phase in the alloy ZX7. However, formation of the latter compound was dependent on solidification conditions and in the alloy ZX3 (no Al present); it is likely that the C14-NbCr_2_ Laves phase participated in a ternary eutectic (see discussion above).

#### 4.2.3. Composition of Phases

The solubilities of Si and Sn in the Nb_ss_ were higher when only Cr was present in the alloys (compare data in Table S2 with Tables S3 and S4 in the Supplemental data) but the Si/Sn ratio did not change significantly (Table 6). In the alloys ZX5 and ZX7 the Si + Al + Sn concentration in the Nb_ss_ was constant and increased slightly in the Ti-rich Nb_ss_ when Al and Cr were simultaneously present in the alloy, see Table 6.

In the cast alloys the additions of Al and Cr individually or simultaneously did not affect the solubility of Sn in the Nb_5_Si_3_. The Al substituted Si in the Nb_5_Si_3_ and in the Al containing alloys the Si + Al + Sn concentration in the Nb_5_Si_3_ was slightly lower than the Si concentration in the unalloyed Nb_5_Si_3_ (36.5 at.%). This effect of Al on the Nb_5_Si_3_ has been reported before for the non-Sn-containing alloy Nb-24Ti-5Al-5Cr (alloy KZ5 in a previous work [16]).

The additions of Cr and Al individually or simultaneously did not affect the solubility of Sn in the Nb_ss_ + Nb_5_Si_3_ eutectic (Tables S2–S4 in the Supplemental data) but Cr and Al had weak and strong effect on the solubility of Si in the eutectic, respectively, with the effect of Al linked with its effect on the Si concentration in Nb_5_Si_3_ (see Si + Sn and Si + Sn + Al data for the eutectic in Table 6). In the cast alloys containing Al the Nb_ss_ + Nb_5_Si_3_ eutectic composition was in the range of values reported for the metastable Nb_ss_ + βNb_5_Si_3_ eutectic [13].

In the heat-treated alloys the addition of Cr or Al individually did not affect the solubility of Sn in the Nb_ss_, but the simultaneous addition of these elements reduced it (compare Tables S2 and S3 with Table S4 in the Supplemental data). In all the heat-treated alloys the solubility of Si in the Nb_ss_ was reduced compared with the cast condition, which is in agreement with the literature, and consequently the Si/Sn ratio and the Si + Sn + Al sum were reduced to approximately 0.3 and 9 at.%, respectively (see Table 6).

### 4.3. Oxidation

Compared with the Sn free alloys JG1, JG2, JG3, and JG4 [18], of nominal compositions Nb-18Si-5Al-5Cr-5Mo, Nb-24Ti-18Si-5Al-5Cr-5Mo, Nb-24Ti-18Si-5Al-5Cr-2Mo, and Nb-24Ti-18Si-5Al-5Cr-5Hf-2Mo, respectively, at 800 °C the linear oxidation rate constants of the alloys ZX3, ZX5, and ZX7 (Table 2) were at least one order of magnitude lower and the parabolic oxidation rate constants were of the same order. Compared with the cast Sn containing alloy JG6 [9] of nominal composition Nb-24Ti-18Si-5Al-5Cr-5Hf-5Sn-2Mo, the parabolic rate constants of the alloy ZX5 and ZX7 were slightly higher. Compared with the same alloys, at 1200 °C the linear oxidation rate constants of the alloys ZX3 and ZX7 were of the same order but that of the alloy ZX5 was one order of magnitude lower (Table 2 [9,18]) and the parabolic rate constants of all three alloys were two orders of magnitude lower than those of the alloys JG1, JG2, JG3, and JG4 [18] and of the same order with alloy JG6 [9].

The oxide scale formed on the alloy ZX3 at 800 °C adhered to the substrate. This was not the case in the alloy KZ4 [17] which contained no Sn (the nominal composition of the alloy KZ4 was Nb-24Ti-18Si-5Cr). In other words, the 2 at.% Sn addition to the alloy ZX3 improved the adhesion of the scale compared with KZ4 and this was attributed to Cr strengthening the synergy of Ti and Sn in improving oxidation at 800 °C, i.e., in the pest regime. Improvements in the adhesion of the scale were also observed with the alloys ZX5 and ZX7 (compare figure S5 in the Supplemental data with Figure 2 in a previous work [17]). At 1200 °C, the oxide scale formed on all three alloys spalled off from the substrate, as reported for most of the studied Nb silicide-based alloys with/without Sn [10,17,18].

At both temperatures the scales formed on all three alloys consisted of Nb-rich and Nb and Si-rich oxides and Ti-rich oxide also was formed in the scales of the alloys ZX3 and ZX7 at 1200 °C (Table 3 and Table 4). Furthermore, at both temperatures Sn-rich layers/zones with Sn-based intermetallic phases were observed in the substrate at the scale/substrate interface. In the Sn-rich zones formed at 1200 °C the presence of Sn-based intermetallics was confirmed (Table 4). In the substrate the solid solution and intermetallic phases were contaminated by oxygen, the former more severely than the latter and the contamination decreased with distance from the scale/substrate interface and both phases were contaminated in the bulk of the alloys. These results and observations are discussed below.

The Nb-rich oxides formed on the alloys ZX3, ZX5, and ZX7 at 800 and 1200 °C are compared in the Table S12 in the Supplemental data. The average oxygen concentration in the oxides was close to that in NbO_2_ (66.7 at.%) and Nb_2_O_5_ (71.4 at.%). These oxides could be considered to be mixtures of Ti_2_Nb_10_O_29_ and TiNb_2_O_7_ oxides [29]. The latter are most commonly identified in the oxide scales formed on Nb-silicide-based alloys [2,17,18]. The concentrations of elements in the Nb-rich oxide formed on the alloy ZX7 at 1200 °C were similar to those reported for the same oxide for the alloy JG6 at the same temperature [9]. The (Nb + Ti)/(Al + Sn) ratio was higher in the alloy ZX7 at both temperatures and increased at 1200 °C compared with 800 °C. The Nb, Ti, Al, and O concentrations and the Nb/Ti ratio were essentially the same in the Nb-rich oxide formed on the alloy ZX5 at both temperatures. In the case of the alloys ZX3 and ZX7, the Nb and Ti concentrations increased and decreased, respectively, the Nb/Ti ratio increased at 1200 °C compared with 800 °C, the (Nb + Ti)/(Cr + Sn) ratio was the same at 800 °C, and the Cr + Sn sum was the same at 800 °C and essentially zero at 1200 °C. The Al + Sn sum in the oxides formed on the alloys ZX5 and ZX7 was reduced at 1200 °C compared with 800 °C and at each temperature was lower in the alloy ZX7. The sum of the other elements excluding Nb and Ti (=OE in Table S12 in the Supplemental data) was higher at 800 °C than 1200 °C and significantly decreased at the latter temperature for the oxide formed on the alloys ZX5 and ZX7. The (Nb + Ti)/(OE) ratio was higher for the alloy ZX5 compared with the alloy ZX3 at both temperatures and significantly increased in the alloy ZX7 at 1200 °C. 

The Nb and Si-rich oxides formed on the alloys ZX3, ZX5, and ZX7 at 800 and 1200 °C are compared in the Table S13 in the Supplemental data. The average oxygen concentration in these oxides was close to that in NbO_2_ (66.7 at.%), SiO_2_ (66.7 at.%), and Nb_2_O_5_ (71.4 at.%). The oxide could be considered as a mixture of Nb_2_O_5_ and SiO_2_ oxides [30]. The latter two oxides have been reported in the scales of Nb silicide-based alloys [18]. Within experimental error, the oxides had the same Nb, Ti, Cr, Al, and Sn concentrations in all three alloys at both temperatures. For the alloys ZX3 and ZX5 the Nb + Ti sum and the [Nb + Ti]/Si and [Nb + Ti/[Si + Sn] ratios were highly similar at each temperature. For the alloy ZX7, the Nb/Ti and [Nb + Ti]/[Al + Cr + Sn] ratios were the same at both temperatures and for the latter alloy the sum of the other elements excluding Nb and Ti (=OE, see Table S13 in the Supplemental data) and the [Nb + Ti]/[OE] and [Nb + Ti]/[Cr + Sn] ratios were the same at both temperatures.

The Ti-rich oxides formed on the alloys ZX3 and ZX7 at 1200 °C are compared in the Table S14 in the Supplemental data. Ti-rich oxide was not found in the scales formed at 800 °C and also was not found in the scale formed on the alloy ZX5 at 1200 °C, which would suggest that Cr promoted the formation of this oxide only at 1200 °C. The average oxygen concentration in the oxides was close to that in NbO_2_ (66.7 at.%), TiO_2_ (66.7 at.%), and Nb_2_O_5_ (71.4 at.%) and could be considered to be mixtures of TiO_2_ and TiNb_2_O_7_ oxides [29]. The latter oxides have been identified in the scales formed on Nb-silicide-based alloys [2,17,18]. The concentrations of elements in the oxide formed on the alloy ZX7 at 1200 °C were similar to those reported for the same oxide for the alloy JG6 at the same temperature [9]. The Sn concentration and the Nb + Ti sum were the same in the alloys ZX3 and ZX7.

The chemical compositions of the Nb_ss_ in the bulk microstructures of the alloys after isothermal oxidation at 800 °C and 1200 °C are compared in the Table S15 in the Supplemental data. The oxygen concentration at 1200 °C was higher (i.e., more severe contamination). The Sn concentrations were similar to those in the cast and heat-treated alloys, the Cr concentration was the same at both temperatures and within the range of values for the cast and heat-treated alloys, the Al concentration was lower than those in the cast and heat-treated alloys, the Ti concentrations at 800 °C were within the ranges of the cast alloys and lower at 1200 °C, and the Si concentrations at 800 °C were lower than in the cast alloys and at 1200 °C slightly lower than those in the heat-treated alloys. The solid solution had similar Si/Sn ratios at 800 °C for the three alloys and at 1200 °C for the alloys ZX3 and ZX7. Essentially, the Al + Si + Sn sum was the same in the alloys ZX5 and ZX7 at 800 °C. The alloy ZX3 had similar Nb/Ti, OE, Cr + Sn and Ti/[OE] at both temperatures.

The chemical compositions of the Nb_5_Si_3_ in the bulk microstructures of the alloys after isothermal oxidation at 800 °C and 1200 °C are compared in Table S16 in the Supplemental data. The concentrations of Al, Cr, Si, Sn, and Ti were essentially the same as in the cast alloys (compare Tables S2–S4 with Table S16 in the Supplemental data). The Nb_5_Si_3_ was contaminated by oxygen in the bulk of all three alloys and at both temperatures and as result the Nb concentration in the Nb_5_Si_3_ was reduced. The contamination by oxygen was more severe at 1200 °C and was higher for the alloy ZX7 and lower for the alloy ZX3 at both temperatures (see arrows in Table S16 in the Supplemental data). At both temperatures the concentration of Sn in the Nb_5_Si_3_ was low and was the same in the alloys ZX3 and ZX5; like the Nb/Ti ratio and the Si + Sn sum. The concentration of Al in the Nb_5_Si_3_ was lower at 1200 °C and at each temperature the Al + Si + Sn sums were the same in the alloys ZX5 and ZX7.

The chemical compositions of the A15-Nb_3_X intermetallic in the bulk microstructures of the alloys ZX5 and ZX7 after isothermal oxidation at 1200 °C are compared in the Table S17 in the Supplemental data. The contamination of the A15 intermetallic was in between those of the Nb_ss_ and the Nb_5_Si_3_ and was more severe in the alloy ZX7. The concentrations of Al, Cr, Si, Sn, and Ti were essentially the same as in the heat-treated alloys (compare Tables S3 and S4 with Table S17 in the Supplemental data). It should be noted that A15 intermetallics were not observed in the microstructures of the cast alloys but only after the heat treatment at 1500 °C and 1450 °C, for alloys ZX5 and ZX7, respectively. Compared with the Nb_ss_, the Nb/Ti ratio and the Si + Sn and Al + Si + Sn sums of A15-Nb_3_X were higher (Tables S15 and S17 in the Supplemental data). However, the composition of the A15-Nb_3_X observed in the bulk of the oxidised specimens was different from the A15 compounds that were observed in the Sn-rich zones formed at 1200 °C, where the data indicated the formation of A15-Nb_3_Sn (see Tables S8 and S9 in the Supplemental data).

#### 4.3.1. Tin Rich Areas

Geng et al. [9] in their study of the effect of Sn on the oxidation of Nb silicide-based alloys suggested that the Sn addition encouraged the oxidation resistance of the Nb_ss_ and thus improved the oxidation behaviour of the alloys compared with the non-Sn-containing alloys. No evidence was provided for the Sn-rich layer formed at 800 °C in the area between the scale and substrate when 5 at.% of Sn was added in their alloy JG6 (nominal composition Nb-24Ti-18Si-5Al-5Cr-5Hf-5Sn-2Mo). Their comment for 800 °C was based on evidence for a Sn-rich phase that formed below the scale during oxidation at 1200 °C. Knittel et al. [10] carried out a study of the oxidation of alloys with compositions based on the MASC alloy (see above) with various Sn additions. In their work, also a Sn-rich layer was reported, and the authors suggested that this Sn-rich layer acted as a diffusion barrier against oxygen and contributed to eliminate pest oxidation.

The Sn-rich layer formed at the 800 °C oxidation of the alloys ZX3 was very thin and thus difficult to identify its composition using WDS. Vellios and Tsakiropoulos [12] also provided evidence for the formation of a Sn-rich layer at the scale substrate interface during oxidation at 800 °C. Furthermore, the research presented in this paper confirmed that the Sn-rich area zone did not stop the diffusion of oxygen to the bulk.

At 1200 °C a Sn-rich zone was formed in the substrate below the scale instead of the Sn-rich layer formed at 800 °C (compare Figure 4 with Figure 5a,b). Geng et al. [9] were the first researchers to report enrichment with Sn of the diffusion zone below the scale in their Sn containing alloy JG6 (see above) that was oxidised at 1200 °C for 100 h and, based on their WDS data, suggested that Sn-rich intermetallics like Nb_3_Sn and Nb_5_Sn_2_Si were most likely present in the Sn-rich regions. Knittel et al. [10] also reported that a Sn-rich area formed after oxidation at 1100 °C and that its thickness increased with the concentration of Sn in the alloy.

In the alloy ZX3, the WDS data would suggest the presence of Nb_3_Sn, Nb_5_Sn_2_Si, and NbSn_2_, all of which were contaminated by oxygen (Table S8 in the Supplemental data). According to Figure 5b, Sn-rich areas surrounded the Nb_5_Si_3_ (which was contaminated by oxygen, Table S8 in the Supplemental data) and were formed in the areas that were occupied by the Nb_ss_ in the cast and heat-treated alloy, see Figure 1c. It is suggested that as the surface regions became enriched with Sn (the reasons why this happens will be discussed below) the Nb_3_Sn formed in the Sn-rich zone in the case of the alloy ZX3 even though this A15 compound was not observed in the cast and the head treated microstructures.

In the case of the alloy ZX5, at 1200 °C, a Sn-rich zone formed at the scale/substrate interface, with thickness about 10 µm (Table 4). In the Sn-rich zone the Nb_3_Sn was observed with Si + Al + Sn = 25.5 at.%, Si/Sn = 0.06, and (Si + Al + Sn)/O_2_ = 3.7 (Table S9 in the Supplemental data). The Nb_3_Sn in the Sn-rich zone was poorer in Al, Si, and Ti and richer in Sn compared with the bulk of the oxidised specimen where the A15-Nb_3_X had Si + Al + Sn = 15.4 at.%, Si/Sn = 0.6, and (Si + Al + Sn)/O_2_ = 2.6 (Table S9 in the Supplemental data). It should be noted that in the bulk of the alloy ZX5 that was heat-treated at 1500 °C the A15-Nb_3_X had Si + Al + Sn = 18.8 at.% and Si/Sn = 0.9.

In the case of the alloy ZX7, at 1200 °C, the Sn-rich zone was noticeably thicker near corners (Figure 7a and Table 4). In the Sn-rich zone, the Sn concentration varied. Some of the Nb_5_Si_3_ grains exhibited substructure and second phase Sn-rich particles (Figure 7b,c, analysis number 10 in Table S11 in Supplemental data). Similar Sn-rich features were also observed in the Nb_5_Si_3_ in the bulk. Sn-rich phases in the Sn-rich zone included the intermetallics NbSn_2_, Nb_5_Sn_2_Si, and Nb_3_Sn and these intermetallics were contaminated by oxygen, as was the Nb_5_Si_3_.

##### Surface Segregation of Sn

The compositions of the phases in the bulk of the oxidised specimens, when compared with those of the cast and/or heat-treated alloys (see discussion earlier in this section), would suggest that the Nb_ss_ contributed the elements that formed the oxides that were observed in the scales, particularly the solid solution in the diffusion zone. Furthermore, comparison of the data for the types of oxides that were observed in the scales formed on the alloys ZX3, ZX5, and ZX7 at 800 °C and 1200 °C shows that the concentration of Sn was below 1 at.% or essentially zero, particularly at 1200 °C for all alloys. In other words, even though Sn segregated in the substrate in areas near the scale/substrate interface where it formed Sn-rich layers or zones, it did not participate in the oxides forming the scales at both temperatures.

A common feature of all the alloys of this research was the formation of Sn-rich areas at the scale/substrate interface, the thickness of which increased with oxidation temperature, making the analysis of Sn-rich phases possible in the alloys oxidised at 1200 °C. In the Sn-rich areas, different Sn-rich intermetallics were formed at the scale/substrate interface. These intermetallics were NbSn_2_, Nb_5_Sn_2_Si, and Nb_3_Sn, the former two with Ti, Al, and Cr and the latter with Ti, Cr, Al, and Si in their composition. These Sn-rich compounds were contaminated by oxygen (Tables S5–S11 in the Supplemental data).

Surface segregation of solute elements in an alloy was predicted by Gibbs [31] who stated that the equilibrium chemical composition of the surface of an alloy should not necessarily be the same as its bulk chemical composition. Experimental research has confirmed surface segregation. Almost all the research has concentrated on binary systems. There is limited research on ternary systems.

The research on binary systems confirmed that the segregation of an element to the surface depends (i) on bulk composition, (ii) temperature, (iii) surface orientation, and (iv) the presence of grain boundaries in a polycrystalline alloy. From (iii) and (iv) above it follows that since the grains near the surface would be of different orientations, differences in the extent (magnitude) of surface segregation should be expected between different grains. Research on ternary alloys with at least two surface active solute elements has also indicated (v) co-segregation (meaning synergistic segregation) which was observed in systems with strong attractive interaction of the solutes or (vi) site competition which was observed when the surface became saturated by surface active solutes and occurred when the surface active solutes attracted or repelled each other [32]. If we take the surface segregation of Sn for granted, then the observations referred to in the previous paragraph do not contradict any of (i)–(v) above. But why does Sn segregate to a surface?

Gibbs [31] showed that the enrichment of a surface in a solute element (i.e., the excess surface concentration of a solute) over its bulk concentration depends on the composition dependence of the surface tension and that a solute element should segregate to the surface if the surface tension decreases with increasing concentration of the solute. Such data about the surface tension is not available for most alloys.

Application of the theory of Gibbs to calculate the excess surface concentration is hindered by the lack of data. Thus, different “theories” have been proposed to account for surface segregation of solutes. These include (a) an approach based on the heat of sublimation according to which the solute in the alloy with lower heat of sublimation should segregate to the surface (we shall call this “theory A”) [33], (b) an approach based on elastic strain energy according to which the larger the solute atom relative to the solvent the higher the degree of surface segregation (we shall call this “theory B”) [34], (c) an approach based on bulk alloy phase diagrams [35] according to which the surface segregation is related with the partitioning of solute to the melt of the alloy, and surface segregation should occur when, owing to distribution (partitioning) of solute, the melt is richer in solute than the solid (we shall call this “theory C”), and (d) an approach based on surface energy according to which the element with the lower surface energy segregates [36] (we shall call this “theory D”).

In addition to aforementioned “theories” A to D, electronic theory and computer simulations have been used to predict surface segregation of solutes in different solvents, particularly for transition metals—transition metal binary alloys, see the literature [32,36,37,38,39,40]. We shall refer to the results of electronic theory calculations and computer simulations as “theory E”.

Considering “theory A”, the heat of sublimation (kJ/mol) of solvent and solute elements of binary Nb-X alloys with X the solutes of this research is as follows Nb—689.9, Ti—425, Si—359, Cr—339.5, Sn (white)—296.1, and Al—284. Thus, according to “theory A” all the solutes in the alloys of this research should segregate to the surface of Nb-X alloys. Considering “theory B”, the atomic size (Å) of solvent and solute elements of binary Nb-X alloys with X the solutes of this research is as follows Nb—1.429, Ti—1.462, Si—1.153, Cr—1.249, Al—1.432, and Sn—1.620. Thus, according to “theory B” the solutes Ti, Al, and Sn should segregate to the surface of Nb-X alloys. Considering “theory C”, surface segregation should be expected for the binaries Nb-Si, Nb-Sn, and Nb-Cr (because of small freezing range) but not for Nb-Ti and Nb-Al.

Considering “theory D”, the surface energy (F_S_) data from Murr [41] predicts “strong” surface segregation for Al and Sn. If we were to use the equation F_s_ = 1.2 γ_LV_ + 0.45 (T_m_ − T) in Murr [41] with γ_LV_ data from Table 3.4 in Murr [41] we calculate F_s_ values according to “theory D”, then the solutes Ti, Si, Al, Cr, and Sn should segregate to the surface, with “strong” segregation for Sn, Si and Al. These conclusions are also supported by another past work [36]. Furthermore, data about surface energies from a previous paper [42] would also predict (according to “theory D”) surface segregation for Ti, Si, Al, Cr, and Sn, with “strong” segregation for Sn, Si, and Al. The equation 10 and data from Table 1 from a previous paper [42] show that the surface concentration of Sn in Nb-5Sn would be 6.4 at.%.

Considering “theory E”, Ruban et al. [40] predicted surface segregation of Ti in Nb-Ti, while Mukherjee and Moran-Lopez [38] predicted surface segregation of Ti and Cr. However, Christensen et al. [39] did not predict surface segregation of Ti.

In summary, the elements predicted to segregate to the surface of binary Nb-X alloys are given in Table 7. All proposed approaches for surface segregation confirm that Sn should segregate to the surface, and would suggest that the other solute elements in the alloys of this study could also segregate to the surface.

Opila [43] showed (i) that the surface segregation of Sn in a polycrystalline dilute Pd-Sn alloy was enhanced in the presence of an oxidising atmosphere and (ii) that the surface segregation near g.bs was also enhanced. The predictions for surface segregation (Table 7), the experimental results of Opila, the fact that Al, Si, and Sn can form equilibrium A15 (Nb_3_Al, Nb_3_Sn) and metastable A15 (Nb_3_Si) compounds, and that Nb_5_Sn_2_X can form with X = Al, Si can explain (a) the formation of Sn-rich areas at the scale/substrate interface and (b) the formation of different Sn-rich intermetallics at the scale/substrate interface depending on the surface concentration of Sn and other solute(s).

### 4.4. Summary and Conclusions

The alloys of nominal compositions Nb-24Ti-18Si-5Cr-2Sn (ZX3), Nb-24Ti-18Si-5Al-2Sn (ZX5), and Nb-24Ti-18Si-5Al-5Cr-2Sn (ZX7) were studied in the as-cast and heat-treated conditions and after isothermal oxidation in air at 800 and 1200 °C for 100 h. The findings and conclusions of the research are as follows.

1. There was macrosegregation of Si and Ti in the alloys ZX3 and ZX5 and only of Si in the alloy ZX7. The synergy of Al and Sn had the strongest effect on the increase of the macrosegregation of Si.
2. Nb_ss_ was stable in all alloys. Tin and Ti exhibited opposite partitioning behaviour in the Nb_ss_.
3. βNb_5_Si_3_ was present in the microstructures of all three cast alloys and had partially transformed to αNb_5_Si_3_ in the alloy ZX3. Aluminium in synergy with Sn increased the sluggishness of the βNb_5_Si_3_ to αNb_5_Si_3_ transformation during solidification. After the heat treatment the transformation of βNb_5_Si_3_ to αNb_5_Si_3_ had been completed in all three alloys. Fine Nb_ss_ precipitates from the βNb_5_Si_3_ → Nb_ss_ + αNb_5_Si_3_ transformation were observed inside some αNb_5_Si_3_ grains in the alloys ZX5 and ZX7.
4. In the alloys ZX5 and ZX7 the A15-Nb_3_X (X = Al, Si, Sn) formed after the heat treatment. Therefore, the synergy of Al and Sn can promote the stability of A15-Nb_3_X intermetallic in Nb-silicide-based alloys even at low Sn concentration.
5. A Nb_ss_ + Nb_5_Si_3_ eutectic was observed in all three alloys with composition close to that of the metastable eutectic and there was evidence of anomalous eutectic in the parts of the alloys ZX3 and ZX7 that had solidified under high cooling rates and/or high melt undercooling. A very fine ternary Nb_ss_ + Nb_5_Si_3_ + NbCr_2_ eutectic was also observed in parts of the alloy ZX3 that had solidified under high cooling rate.
6. At 800 °C none of the alloys suffered from catastrophic pest oxidation. The smaller oxidation rate constant was exhibited by the alloy ZX7. A thin Sn-rich layer formed continuously between the scale and Nb_ss_ in the alloys ZX3 and ZX5.
7. At 1200 °C the scales formed on all three alloys spalled off, the alloys exhibited parabolic oxidation in the early stages followed by linear oxidation and the alloy ZX5 gave the smaller rate constant values. A thicker continuous Sn-rich zone formed between the scale and substrate in all three alloys. This zone was noticeably thicker near the corners of the specimen of the alloy ZX7 and continuous around the whole specimen. The Nb_3_Sn, Nb_5_Sn_2_Si, and NbSn_2_ compounds were observed in the Sn-rich zone. The formation of Sn-rich zone was supported by different theories for surface segregation of solute elements in alloys.
8. At both temperatures the scales formed on all three alloys consisted of Nb-rich and Nb and Si-rich oxides and Ti-rich oxide also was formed in the scales of the alloys ZX3 and ZX7 at 1200 °C. The formation of a Sn-rich layer/zone did not prevent the contamination of the bulk of the specimens by oxygen, as both the Nb_ss_ and Nb_5_Si_3_ were contaminated by oxygen in the bulk of the oxidised specimens, the former more severely than the latter.

## Figures and Tables

**Table 1:** Comparison of phases present in the cast and heat-treated alloys ZX3, ZX5, and ZX7.

Alloy	As-Cast		Heat-Treated	
	Composition	Phases	Composition	1500 °C/Phases
ZX3	Nb-24.9Ti-20.8Si-4.5Cr-2.0Sn	αNb_5_Si_3_, βNb_5_Si_3_, Nb_ss_, (Nb_ss_ + Nb_5_Si_3_)_eutectic_ C14-NbCr_2_ Laves phase	Nb-25.7Ti-19.7Si-4.6Cr-2.1Sn	Nb_ss_, αNb_5_Si_3_
ZX5	Nb-25.1Ti-19.1Si-4.9Al-1.8Sn	βNb_5_Si_3_, Nb_ss_, (Nb_ss_ + Nb_5_Si_3_)_eutectic_	Nb-22.7Ti-20.1Si-4.3Al-1.6Sn	αNb_5_Si_3_, Nb_3_Sn, Nb_ss_, C14-NbCr_2_ Laves phase
	**Composition**	**Phases**	**Composition**	**1450 °C/Phases**
ZX7	Nb-24.7Ti-15.8Si-5.0Cr-5.1Al-2.1Sn	βNb_5_Si_3_, Nb_ss_, (Nb_ss_ + Nb_5_Si_3_)_eutectic_ C14-NbCr_2_ Laves phase	Nb-26.4Ti-14.6Si-5.4Cr-4.6Al-2Sn	αNb_5_Si_3_, Nb_3_Sn, Nb_ss_, C14-NbCr_2_ Laves phase

**Table 2:** The linear k_l_ (g cm^−2^ s^−1^) and parabolic k_p_ (g^2^ cm^−4^ s^−1^) oxidation kinetic rate constants of the alloys ZX3, ZX5, and ZX7 for isothermal oxidation at 800 and 1200 °C.

T (°C)	Alloy		
	ZX3	ZX5	ZX7
800	k_l_ = 1.69 × 10^−8^	k_p_ = 5.16 × 10^−11^ (t ≤ 50 h) k_l_ = 1.80 × 10^−8^ (t > 50 h)	k_p_ = 1.76 × 10^−11^ (t ≤ 41.7 h) k_l_ = 9.94 × 10^−9^ (t > 41.7 h)
1200	k_p_ = 4.54 × 10^−9^ (t ≤ 16.7 h) k_l_ = 1.42 × 10^−7^ (t > 16.7 h)	k_p_ = 1.15 × 10^−9^ (t ≤ 21.7 h) k_l_ = 6.70 × 10^−8^ (t > 21.7 h)	k_l_ = 5.91 × 10^−9^ (t ≤ 36.7 h) k_l_ = 1.42 × 10^−7^ (t > 36.7 h)

**Table 3:** Comparison of scales, diffusion zones, and bulk microstructures in the alloys at 800 °C.

Alloy	Oxide Scale		Enrichment in Sn and Sn-Rich Phase(s) Formation	Oxygen Diffusion Zone		Bulk Microstructure
	Thickness (µm)	Oxides *		Thickness (µm)	Phases *	Phases *
ZX3	15	Nb-rich, Nb and Si-rich	Yes	40	Nb_5_Si_3_, oxidised Nb_ss_	Nb_5_Si_3_, Nb_ss_
ZX5	10–15	Nb-rich, Nb and Si-rich	Yes	50	Nb_5_Si_3_, oxidised Nb_ss_	Nb_5_Si_3_, Nb_ss_
ZX7	10	Nb-rich, Nb and Si-rich	Not observed	40	Nb_5_Si_3_, oxidised Nb_ss_	Nb_5_Si_3_, Nb_ss_

* Chemical compositions data in Tables S5–S7 in the Supplemental data.

**Table 4:** Comparison of scales, Sn-rich zone, and bulk microstructures of the alloys at 1200 °C.

Alloy	Scale		Sn-Rich Zone		Bulk
	Thickness (µm)	Oxides *	Thickness (µm)	Sn-Rich Intermetallics *	Phases *
ZX3	450	Nb-rich, Nb and Si-rich, Ti-rich	25	Nb_5_Si_3_, Nb_3_Sn, Nb_5_Sn_2_Si, NbSn_2_	Nb_5_Si_3_, Nb_ss_, Laves phase
ZX5	300	Nb-rich, Nb and Si-rich,	10	Nb_5_Si_3_, Nb_3_Sn, Nb_5_Sn_2_Si	Nb_5_Si_3_, Nb_ss_, A15-Nb_3_X,
ZX7	400	Nb-rich, Nb and Si-rich, Ti-rich	35	Nb_5_Si_3_, NbSn_2_, Nb_5_Sn_2_Si	Nb_5_Si_3_, Nb_ss_, A15-Nb_3_X, Laves phase

* Chemical compositions data in Tables S8–S11 in the Supplemental data.

**Table 5:** Alloy parameters of the macrosegregation of Si in the cast alloys of this study and in the reference alloys KZ4, KZ5 and KZ7.

Alloy	ΔH_m_ kJ/mol	T_m_ (K)	ΔH_m_/T_m_ J/mol K	ΔH_m_^sd^/ΔH_m_^sp^	T_m_^sd^ (K)	T_m_^sp^ (K)	T_m_^sd^/T_m_^sd^	(ΔH_m_/T_m_) × (ΔH_m_^sd^/ΔH_m_^sp^)^−1^	MACSi (at.%)
ZX3	28.97	2257	12.84	1.738	1896	361	5.25	7.39	3.6
KZ4	28.20	2335	12.1	2.44	2060	275	7.5	4.96	1.9
ZX5	28.1	2215	12.68	1.74	1837	377	4.87	7.29	5.5
KZ7	27.7	2272	12.19	2.15	1948	324	6	5.67	2.3
ZX7	29.45	2214	13.53	1.79	1889	325	5.82	7.56	3
KZ5	27.50	2239	12.28	2.05	1909	330	5.78	5.99	1.3

Nominal compositions: KZ4 = Nb-24Ti-18Si-5Cr, KZ5 = Nb-24Ti-18Si-5Al-5Cr, KZ7 = Nb-24Ti-18Si-5Al, data for these alloys can be found in the literature [16].

**Table 6:** Comparison of Nb_ss_, Ti-rich Nb_ss_, Nb_5_Si_3_, Nb_ss_ + Nb_5_Si_3_ eutectic, and A15 phases in the alloys ZX3, ZX5, and ZX7.

Alloy and Condition	Si + Sn (at.%)					Si + Sn + Al (at.%)					Si/Sn		
	Nb_ss_	Ti-Rich Nb_ss_	Nb_ss_ + Nb_5_Si_3_ Eutectic	Nb_5_Si_3_	A15-Nb_3_X	Nb_ss_	Ti-Rich Nb_ss_	Nb_ss_ + Nb_5_Si_3_ Eutectic	Nb_5_Si_3_	A15-Nb_3_X	Nb_ss_	Ti-Rich Nb_ss_	A15-Nb_3_X
ZX3-AC	6.5	6.1	17.5	36.7							0.9	1.1	
ZX5-AC	5.2	5.2	14.7	31.7		11.1	11.1	19.8	35.4		1	0.9	
ZX7-AC	5.1	6.2	14.6	31.1		11	12.3	19.8	35.4		0.8	1.3	
ZX3-HT	4.1			37							0.2		
ZX5-HT	2.6			34.5	10.4	9.1			36.8	18.8	0.3		0.9
ZX7-HT	2.4			35.2	9.7	9.2			36.9	18.6	0.3		0.9

**Table 7:** Solutes in Nb-X segregating to the surface.

Theory	Surface Segregating Element				
A	Al	Cr	Si	Sn	Ti
B	Al			Sn	Ti
C		Cr	Si	Sn	
D	Al	Cr	Si	Sn	Ti
E *		Cr			Ti?

* For transition metal–transition metal binary alloys, see text.

## Supplementary Materials

The following are available online at http://www.mdpi.com/1996-1944/11/10/1826/s1, Figure S1: X-ray diffractograms of the cast and heat treated alloys (a,b) ZX3, (c,d) ZX5, (e,f) ZX7, Figure S2: BSE image and X-ray maps showing the presence of a Cr rich phase (assumed to be the C14-NbCr2 Laves phase) in the bottom of the button of the cast alloy ZX3, Figure S3: Top left, BSE image of the microstructure in the bottom area of the button of the cast alloy ZX3 showing areas with eutectic. Top right, BSE image of eutectic observed in the bottom of the button. X-ray elemental maps for the top right image, Figure S4: BSE image and X-ray elemental maps of the bottom area of the cast alloy ZX7, Figure S5: The oxidised specimens at 800 °C (a,c,e) and at 1200 °C (b,d,f). Images correspond to alloys as follows: (a,b) ZX3, (c,d) ZX5 and (e,f) ZX7, Figure S6: BSE image and X-ray elemental maps of the Sn rich zone of the alloy ZX7 after oxidation at 1200 °C, Table S1: Calibration standards and their compositions for the EPMA analyses, Table S2: EDS analysis (at.%) of the as cast and heat treated alloy ZX3, Table S3: EDS analysis (at.%) of the as cast and heat treated alloy ZX5, Table S4: EDS analysis (at.%) of the as cast and heat treated alloy ZX7, Table S5: WDS analysis (at.%) of oxides in the scale and phases in the bulk of the alloy ZX3 at 800 °C, Table S6: WDS analysis data (at.%) of oxides in the scale, and phases in the diffusion zone and bulk of the ZX5 after isothermal oxidation for 100 h at 800 °C, Table S7: WDS analysis data (at.%) of oxides in the scale, and phases in the diffusion zone and bulk of the alloy ZX7 after isothermal oxidation for 100 h at 800 °C, Table S8: The WDS analysis data (at.%) of oxides in the scale, and phases in the Sn rich zone and bulk of the alloy ZX3 after isothermal oxidation for 100 h at 1200 °C, Table S9: The WDS analysis data (at.%) of oxides in the scale, and phases in the Sn rich zone and bulk of the alloy ZX5 after isothermal oxidation for 100 h at 1200 °C, Table S10: WDS analysis data (at.%) of the oxides in the scale of the alloy ZX7, Table S11: WDS analysis data (at.%) of the phases in the Sn rich zone and below it towards the bulk in the alloy ZX7 oxidised at 1200 °C, Table S12: Comparison of the Nb rich oxides formed in the scales of the alloys at 800 °C and 1200 °C. The average concentrations of elements are in at.%. OE = other elements excluding Nb and Ti, Table S13: Comparison of the Nb and Si rich oxides formed in the scales of the alloys at 800 °C and 1200 °C. The average concentrations of elements are in at.%. OE = other elements excluding Nb and Ti, Table S14: Comparison of the Ti rich oxides formed in the scales of the alloys ZX3 and ZX7 at 1200 °C. The average concentrations of elements are in at.%. OE = other elements excluding Nb and Ti, Table S15: Comparison of the compositions of the Nbss in the bulk of the oxidised alloys at 800 °C and 1200 °C. There is no data for the Nbss in the alloy ZX7. The average concentrations of elements are in at.%. OE = other elements excluding Nb and Ti, Table S16: Comparison of the compositions of the Nb5Si3 in the bulk of the oxidised alloys at 800 °C and 1200 °C. The average concentrations of elements are in at.%, Table S17: Comparison of the compositions of the A15-Nb3X in the bulk of the alloys ZX5 and ZX7 at 1200 °C. The average concentrations of elements are in at.%.
