# Supplementary material for: A Study of the Effect of 2 at.% Sn on the Microstructure and Isothermal Oxidation at 800 and 1200 °C of Nb-24Ti-18Si-Based Alloys with Al and/or Cr Additions
Source: Materials (Basel). 2018 Sep 25;11(10):1826. doi: 10.3390/ma11101826 (PMC6212920; doi:10.3390/ma11101826)
Supplement: Supplementary file 1 [file materials-11-01826-s001.pdf]

Table S1 Calibration standards and their compositions for the EPMA analyses.

Elements	Reference materials	Composition of the reference materials (wt.%)
Nb	Nb	Nb: 100%
Ti	Rutile	Ti: 59.34%, O: 39.89%, Mg: 0.01%, Fe: 0.59%, Nb: 0.17%
Si	Zircon	Si: 15.24%, Mn: 0.03%,Zr: 49.14%, Hf: 0.82%, O: 34.77%
Al	Spinel	Mg: 17.08%, Al: 37.93%, O:44.99 %
Cr	Cr	Cr: 100%
Sn	Sn	Sn: 100%
O	Forsterite	O:45.5%, Mg:34.5%, Si:20.0%

Table S2 EDS analysis (at. %) of the as cast and heat treated alloy ZX3

Condition/phase	Nb	Ti	Si	Cr	Sn
As cast					
Nb_{ss}	63.7 ± 2.1<br>*61.1 - 66.9*	24.7 ± 1.7<br>*21.5 - 26.9*	3.1 ± 0.7<br>*2.3 - 4.3*	5.1 ± 0.5<br>*4.0 - 5.5*	3.4 ± 0.5<br>*2.7 - 4.3*
Ti-rich Nb_{ss}	43.9 ± 5.5<br>*36.1 - 48.8*	37.0 ± 3.5<br>*34.3 - 41.9*	3.2 ± 1.0<br>*1.9 - 4.1*	13.0 ± 1.7<br>*11.0 - 14.8*	2.9 ± 0.3<br>*2.6 - 3.3*
Nb_5Si_3	44.8 ± 1.7<br>*41.2 - 46.2*	17.8 ± 1.5<br>*16.8 - 20.9*	35.9 ± 0.4<br>*35.3 - 36.7*	0.7 ± 0.4<br>*0.2 - 1.2*	0.8 ± 0.2<br>*0.5 - 1.0*
Eutectic	53.0 ± 2.1<br>*50.5 - 55.5*	24.9 ± 1.4<br>*23.3 - 27.0*	15.1 ± 1.9<br>*12.8 - 17.9*	4.6 ± 0.7<br>*4.0 - 5.7*	2.4 ± 0.3<br>*1.9 - 2.8*
Heat treated					
Nb_{ss}	55.0 ± 0.2<br>*54.7 - 55.4*	31.8 ± 0.2<br>*31.4 - 32.1*	0.7 ± 0.1<br>*0.5 - 0.9*	9.1 ± 0.2<br>*8.9 - 9.4*	3.4 ± 0.1<br>*3.3 - 3.5*
Nb_5Si_3	44.6 ± 0.3<br>*43.9 - 44.8*	18.3 ± 0.5<br>*17.8 - 19.0*	35.8 ± 0.3<br>*35.3 - 36.4*	0.6 ± 0.2<br>*0.4 - 0.8*	0.7 ± 0.1<br>*0.6 - 0.9*
Ti-rich Nb_5Si_3	40.8 ± 0.4<br>*40.0 - 41.6*	21.8 ± 0.4<br>*21.2 - 22.5*	36.4 ± 0.2<br>*36.2 - 36.7*	0.4 ± 0.1<br>*0.3 - 0.6*	0.6 ± 0.1<br>*0.5 - 0.8*

Table S3 EDS analysis (at. %) of the as cast and heat treated alloy ZX5

Condition/phase	Nb	Ti	Si	Al	Sn
As cast					
Nb_{ss}	59.9 ± 0.8<br>*59.0 - 61.0*	29.1 ± 0.9<br>*28.1 - 30.0*	2.6 ± 0.2<br>*2.4 - 2.9*	5.8 ± 0.1<br>*5.7 - 6.0*	2.6 ± 0.1<br>*2.5 - 2.8*
Ti-rich Nb_{ss}	54.9 ± 2.6<br>*50.3 - 57.2*	33.9 ± 2.3<br>*31.8 - 38.1*	2.5 ± 0.2<br>*2.2 - 2.7*	6.0 ± 0.2<br>*5.7 - 6.2*	2.7 ± 0.2<br>*2.5 - 3.0*
Nb_5Si_3	47.7 ± 0.1<br>*47.6 - 48.0*	15.6 ± 0.2<br>*15.3 - 15.9*	33.0 ± 0.3<br>*32.4 - 33.4*	3.0 ± 0.2<br>*2.6 - 3.5*	0.7 ± 0.1<br>*0.6 - 0.8*
Ti-rich Nb_5Si_3	42.0 ± 1.5<br>*39.6 - 44.0*	22.5 ± 1.6<br>*20.2 - 25.1*	30.8 ± 1.0<br>*28.6 - 31.6*	3.7 ± 0.1<br>*3.6 - 4.0*	1.0 ± 0.1<br>*0.8 - 1.1*
Eutectic	57.0 ± 0.9<br>*55.7 - 58.3*	23.2 ± 0.6<br>*22.3 - 24.2*	12.6 ± 0.6<br>*11.5 - 13.5*	5.1 ± 0.3<br>*4.8 - 5.8*	2.1 ± 0.3<br>*1.9 - 2.9*
Heat treated					
Nb_{ss}	60.2 ± 0.2<br>*59.8 - 60.4*	30.7 ± 0.3<br>*30.4 - 31.0*	0.5 ± 0.1<br>*0.4 - 0.8*	6.5 ± 0.3<br>*6.3 - 7.1*	2.1 ± 0.2<br>*1.8 - 2.3*
Nb_5Si_3	46.1 ± 1.5<br>*43.9 - 47.9*	17.2 ± 1.5<br>*15.2 - 19.3*	33.8 ± 0.5<br>*33.0 - 34.6*	2.3 ± 0.4<br>*1.5 - 3.0*	0.6 ± 0.1<br>*0.5 - 0.8*
Nb_3X-A15	58.9 ± 0.4<br>*58.4 - 59.4*	22.4 ± 0.7<br>*21.8 - 23.4*	4.9 ± 0.7<br>*3.9 - 5.6*	8.4 ± 0.5<br>*7.6 - 9.0*	5.4 ± 0.2<br>*5.2 - 5.6*

Table S4 EDS analysis (at. %) of the as cast and heat treated alloy ZX7

Condition & phase	Nb	Ti	Si	Cr	Al	Sn
As cast						
Nb_{ss}	61.7 ± 0.8<br>60.9 - 62.7	22.9 ± 0.5<br>22.3 - 23.3	2.3 ± 0.1<br>2.2 - 2.4	4.4 ± 0.3<br>4.1 - 4.7	5.9 ± 0.2<br>5.7 - 6.1	2.8 ± 0.2<br>2.7 - 3.1
Ti-rich Nb_{ss}	49.4 ± 2.4<br>*47.6 - 52.1*	29.9 ± 1.3<br>*28.5-31.1*	3.5 ± 0.8<br>*2.7 - 4.2*	8.4 ± 0.4<br>*8.0 - 8.6*	6.1 ± 0.1<br>*6.0 - 6.2*	2.7 ± 0.1<br>*2.6 - 2.8*
Nb_5Si_3	45.7 ± 0.4<br>*44.7 - 46.2*	17.7 ± 0.5<br>*17.3 - 18.7*	29.9 ± 0.4<br>*28.9 - 30.2*	1.2 ± 0.2<br>*1.0 - 1.6*	4.3 ± 0.1<br>*4.2 - 4.5*	1.2 ± 0.1<br>*1.1 - 1.3*
Eutectic	48.7 ± 1.5<br>*46.5 - 50.9*	25.9 ± 0.8<br>*24.6 - 27.5*	12.4 ± 1.0<br>*10.7 - 13.7*	5.6 ± 0.4<br>*4.8 - 6.3*	5.2 ± 0.1<br>*5.0 - 5.4*	2.2 ± 0.1<br>*2.1 - 2.3*
Heat treated						
Nb_{ss}	56.7 ± 1.4<br>*55.6 - 59.3*	23.4 ± 0.6<br>*22.6 - 24.4*	0.6 ± 0.2<br>*0.4 - 0.8*	10.7 ± 0.7<br>*9.3 - 11.3*	6.8 ± 0.3<br>*6.4 - 7.2*	1.8 ± 0.3<br>*1.4 - 2.2*
Nb_5Si_3	44.8 ± 0.4<br>*44.3 - 45.4*	17.9 ± 0.3<br>*17.3 - 18.2*	34.5 ± 0.4<br>*34.0 - 34.9*	0.4 ± 0.1<br>*0.3 - 0.6*	1.7 ± 0.2<br>*1.5 - 1.9*	0.7 ± 0.1<br>*0.6 - 0.9*
A15-Nb_3X	57.5 ± 0.9<br>*56.1 - 58.6*	18.4 ± 0.8<br>*17.3 - 19.7*	4.5 ± 1.0<br>*3.4 - 6.3*	5.5 ± 0.3<br>*4.7 - 5.8*	8.9 ± 0.9<br>*7.6 - 9.8*	5.2 ± 0.3<br>*4.9 - 5.6*

Table S5 WDS analysis (at. %) of oxides in the scale and phases in the bulk of the alloy ZX3 at 800 °C.

Phase	Nb	Ti	Si	Cr	Sn	O
**Scale**						
Nb and Si rich oxide	13.9 ± 0.3<br>*13.5 - 14.2*	4.3 ± 0.5<br>*3.9 - 4.8*	11.8 ± 0.9<br>*11.00 - 12.8*	0.3 ± 0.1<br>*0.2 - 0.4*	0.3 ± 0.10<br>*0.2 - 0.4*	69.4 ± 0.3<br>*69.1 - 69.7*
Nb rich oxide	16 ± 1.7<br>*14.2 - 18.2*	9 ± 1.3<br>*7.3 - 10.3*	0.3<br>*0.30 - 0.4*	3.7 ± 1.5<br>*2.1 - 5.6*	0.3 ± 0.2<br>*0.1 - 0.5*	70.7 ± 0.7<br>*70 - 71.6*
**Bulk**						
Nb_{ss}	56.7 ± 4.2<br>*51 - 62.2*	25.9 ± 2.3<br>*22.6 - 28.7*	1.7 ± 0.7<br>*1.3 - 3*	7.2 ± 1.4<br>*5.5 - 9.2*	2.7 ± 0.1<br>*2.7 - 2.9*	5.8 ± 0.6<br>*5.4 - 7.1*
Nb_5Si_3	42.3 ± 0.3<br>*41.8 - 42.6*	16.1 ± 0.3<br>*15.8 - 16.4*	35.8 ± 0.3<br>*35.4 - 36.1*	1<br>*0.90 - 1*	0.7<br>*0.6 - 0.7*	4.2 ± 0.4<br>*3.8 - 4.6*

Table S6 WDS analysis data (at. %) of oxides in the scale, and phases in the diffusion zone and bulk of the ZX5 after isothermal oxidation for 100 h at 800 °C.

Phase	Nb	Ti	Si	Al	Sn	O
**Scale**						
Nb rich oxide	20.1	8.4	1.5	1.8	1.3	66.9
Nb & Si rich oxide	15.5	4.9	12.8	0.5	0.2	66.1
**Diffusion zone**						
Nb_{ss} (in eutectic)	38.7<br>*35.8-40.2*	20.2<br>*18.8-21.7*	1.1<br>*1-1.2*	3.3<br>*3.1-3.5*	1.9<br>*1.8-2*	34.8<br>*33.1-36.1*
Nb_5Si_3	44.9<br>*44.5-45.1*	14.9<br>*14.6-15.1*	34.9<br>*34.3-35.4*	1.8<br>*1.6-2.2*	0.6<br>*0.5-0.7*	2.9<br>*2-3.4*
$Nb_5Si_{3(in\ eutectic)}$	39.6	20.4	33.4	2.5	0.7	3.5
**Bulk**						
Nb_{ss}	54.2	31.8	1.5	4.2	2.6	5.5
Nb_5Si_3	44.7	15	35	1.8	0.6	3

Table S7 WDS analysis data (at. %) of oxides in the scale, and phases in the diffusion zone and bulk of the alloy ZX7 after isothermal oxidation for 100 h at 800 °C.

Phase	Nb	Ti	Si	Cr	Al	Sn	O
**Scale**							
Nb rich oxide	14.2	9.8	1.3	2.8	0.8	1.1	70.00
Nb and Si rich oxide	16.0	5.00	11.8	0.3	0.9	0.3	65.7
**Diffusion Zone**							
Nb_{ss} (average of analyses 28, 32, 34)*	35.4	19	0.8	5.9	4.1	1.9	32.9
Nb_5Si_3 (average of analyses 30, 31, 33)*	41.8	18.5	29.1	1.2	3.4	1.1	4.9
**Bulk**							
Nb_{ss}	51	27.6	1.2	6.2	5.2	2.6	6.2
Nb_5Si_3	43.8	16.8	29.6	1.1	3.7	1.1	3.9

*see text and figure 4f

Table S8 The WDS analysis data (at. %) of oxides in the scale, and phases in the Sn rich zone and bulk of the alloy ZX3 after isothermal oxidation for 100 h at 1200 °C.

Phase	Nb	Ti	Si	Cr	Sn	O
**scale**						
Nb rich oxide	18.3	5.9	4.5	0.4	-	70.9
Ti rich oxide	6.1	17.5	0.2	6.0	0.2	70
Nb & Si rich oxide	13.1	5.5	9.3	0.3	-	71.8
**Sn rich zone**						
$NbSn_2$	31.4	1	0.7	0.3	59.5	7.3
A15-Nb_3Sn	52	15.8	0.3	3	22.8	6
Nb_5Sn_2Si	46.1	13.9	9.3	2.7	24.1	3.9
**bulk**						
Nb_{ss}	57 ± 0.5<br>*56.2 - 57.6*	23.5 ± 0.3<br>*23.3 - 23.9*	0.2<br>*0.1 - 0.2*	7.5 ± 0.1<br>*7.3 - 7.6*	3.3 ± 0.1<br>*3.1 - 3.4*	8.7 ± 0.7<br>*7.7 - 9.6*
Nb_5Si_3	43.9 ± 0.2<br>*43.6 - 44.1*	16.6 ± 0.1<br>*16.5 - 16.8*	34.3 ± 0.7<br>*33 - 34.8*	0.9 ± 0.2<br>*0.5 - 1.2*	0.7<br>*0.6 - 0.8*	3.7 ± 0.3<br>*3.4 - 4.2*

Table S9 The WDS analysis data (at. %) of oxides in the scale, and phases in the Sn rich zone and bulk of the alloy ZX5 after isothermal oxidation for 100 h at 1200 °C.

phase	Nb	Ti	Si	Al	Sn	O
**scale**						
Nb rich oxides	20.7	9.8	0.5	1.8	0.2	67.1
Nb & Si rich oxides	16.3	4.9	10.8	0.5	0.2	67.4
**Sn rich zone**						
A15-Nb_3Sn (average of analyses 15, 16)*	57.7	10	1.4	0.6	23.4	6.9
Nb_5Sn_2Si (analysis 19)	49	10.7	9.7	1.7	22.4	6.5
Nb_5Si_3 (average of analyses 21, 22)*	39.3	19	34.1	2.3	1.9	3.1
**bulk**						
Nb_5Si_3	44.2<br>*43.8-44.6*	14.9<br>*14.8-15.1*	34.6<br>*34.6-34.7*	1.7<br>*1.7-1.7*	0.6<br>*0.5-0.6*	4<br>*3.4-4.6*
Nb_3Al	56.8<br>*54.2-59.1*	22<br>*21.1-23.7*	3.1<br>*2.9-3.5*	7.2<br>*7.1-7.5*	5.1<br>*4.8-5.3*	5.8<br>*4.5-7*
Nb_{ss}	58.8<br>*57.4-62.1*	28<br>*25-30.2*	0.3<br>*0.3- 0.4*	3.4<br>*3.3-3.4*	1.4<br>*1.3-1.5*	8.1<br>*6.1-10.7*

*see text and figure 6c

Table S10 WDS analysis data (at. %) of the oxides in the scale of the alloy ZX7.

Phase/analysis number*	Nb	Ti	Si	Cr	Al	Sn	O
**scale**							
Nb rich oxide	25	5.9	0.1	0.2	0.8	-	68
Ti rich oxide	8.7	15.5	1.3	3.9	3.8	0.2	66.6
Nb & Si rich oxide	14.4	4.5	12.7	0.5	0.7	-	67.2
**Sn rich zone**							
Spot 1	43.4	15.9	30	0.9	3.4	1.6	4.8
Spot 2	43.9	16.2	31.4	0.5	3.3	1.1	3.6
Spot 3	23.4	9.2	0.4	0.4	-	60.9	5.6
Spot 4	41.8	15.9	10.7	2.5	2.1	22.5	4.6

*see text and figure 7e

Table S11 WDS analysis data (at. %) of the phases in the Sn rich zone and below it towards the bulk in the alloy ZX7 oxidised at 1200 °C.

Analysis number*	Nb	Ti	Si	Cr	Al	Sn	O
**Nb_5Si_3**							
Spot 7	43.1	16	30.6	0.6	3.3	1.2	5.2
Spot 8	43.2	16.7	32.1	0.3	2.7	1	4.1
Spot 9	43.8	16.3	31.2	0.5	3	1.5	3.7
Spot 10	46	10.9	28.9	0.8	0.8	7	5.7
Spot 15	41.9	18	33.1	0.3	1.4	0.4	4.9
**Nb_5Si_2Sn**							
Spot 11	42.9	13.4	12	2.7	1.8	19.9	7.3
Spot 12	42.4	14.7	12.8	3	1.7	19.8	5.6
Spot 13	45.6	13.2	6.5	1.2	5.9	21.3	6.3
Spot 14	41.3	16.6	7.5	1.7	5.1	21.6	6.1
**A15 intermetallic**							
Spot 16	55.7	17.3	2.7	4.6	10.1	3.6	6.1
Spot 17	54.8	18.8	2.1	4.7	9	3.3	7.4
**Laves Phase**							
Spot 18	28.4	10	5.8	49.8	2	0.3	3.7

*see text and figure 7d

Table S12. Comparison of the Nb rich oxides formed in the scales of the alloys at 800 °C and 1200 °C.
The average concentrations of elements are in at.%. OE = other elements excluding Nb and Ti

Alloy	Nb	Ti	Si	Cr	Al	Sn	O	Nb+Ti	Al+Sn	Cr+Sn	OE	[Nb+Ti]/[Cr+Sn]	[Nb+Ti]/[Al+Sn]	[Nb+Ti]/[OE]	Nb/Ti
**800 °C**															
ZX3	16	9	0.3	3.7		0.3	70.7	25		4	4.3	6.3		5.8	1.8
ZX5	20.2	8.4	1.5		1.8	1.3	66.8	28.6	3.1		4.6		8.1	6.2	2.4
ZX7	14.2	9.8	1.3	2.8	0.8	1.1	70	24	1.9	3.9	6	6.2	12.6	4	1.5
**1200 °C**															
ZX3	18.3	5.9	4.5	0.4		0	70.9	24.2		0.4	4.9	60.5		4.9	3.1
ZX5	20.7	9.8	0.5		1.8	0.2	67	30.5	2		2.5		15.3	12.2	2.1
ZX7	25	5.9	0.1	0.2	0.8	0	68	30.9	0.8	0.2	1.1	154.5	38.6	28.1	4.2

Table S13. Comparison of the Nb and Si rich oxides formed in the scales of the alloys at 800 °C and 1200 °C.
The average concentrations of elements are in at.%. OE = other elements excluding Nb and Ti

Alloy	Nb	Ti	Si	Cr	Al	Sn	O	Nb+Ti	[Nb+Ti]/Si	Cr+Sn	[Nb+Ti]/[Cr+Sn]	[Nb+Ti]/[Si+Sn]	OE	[Nb+Ti]/[Al+Cr+Sn]	[Nb+Ti]/[OE]	Nb/Ti
**800 °C**																
ZX3	13.9	4.3	11.8	0.3		0.3	69.4	18.2	1.5	0.6	30.3	1.5	12.4		1.5	3.2
ZX5	15.5	4.9	12.8		0.5	0.2	66.1	20.4	1.6			1.6	13.5		1.5	3.2
ZX7	16.1	5	11.8	0.3	0.9	0.3	65.6	21.1	1.8	0.6	35.2	1.7	13.3	14.1	1.6	3.2
**1200 °C**																
ZX3	13.1	5.5	9.3	0.3		0	71.8	18.6	2	0.3	62	2	9.6		1.9	2.4
ZX5	16.3	4.9	10.8		0.5	0.2	67.3	21.2	2			1.9	11.5		1.8	3.3
ZX7	14.4	4.5	12.7	0.5	0.7	0	67.2	18.9	1.5	0.5	37.8	1.5	13.9	15.8	1.4	3.2

Table S14. Comparison of the Ti rich oxides formed in the scales of the alloys ZX3 and ZX7 at 1200 °C. The average concentrations of elements are in at.%. OE = other elements excluding Nb and Ti

Alloy	Nb	Ti	Si	Cr	Al	Sn	O	Nb+Ti	Si+Sn	OE	[Nb+Ti]/[OE]	Nb/Ti
ZX3	6.1	17.5	0.2	6		0.2	70.2	23.6	0.4	6.4	3.7	0.4
ZX7	8.7	15.5	1.3	3.9	3.8	0.2	66.6	24.2	1.5	9.2	2.6	0.6

Table S15. Comparison of the compositions of the Nb_{ss} in the bulk of the oxidised alloys at 800 °C and 1200 °C. There is no data for the Nb_{ss} in the alloy ZX7. The average concentrations of elements are in at.%. OE = other elements excluding Nb and Ti

Alloy	Nb	Ti	Si	Cr	Al	Sn	O	Ti/[OE]	Si+Sn	Si+Sn+Al	Si/Sn	Cr+Sn	OE	Ti+OE	Nb/Ti
**800 °C**															
ZX3	56.7	25.9	1.7	7.2		2.7	5.8	2.2	4.4		0.6	9.9	11.6	37.5	2.2
ZX5	54.4	31.8	1.5		4.2	2.6	5.5	3.8	4.1	8.3	0.6		8.3	40.1	1.7
ZX7	51.3	27.6	1.2	6.2	5.2	2.2	6.3	1.9	3.4	8.6	0.6	8.4	14.8	42.4	1.9
**1200 °C**															
ZX3	56.8	23.5	0.2	7.5		3.3	8.7	2.1	3.5		0.06	10.8	11	34.5	2.4
ZX5	58.8	28	0.3		3.4	1.4	8.1	5.5	3.7	5.1	0.09		5.1	33.1	2.1

Table S16. Comparison of the compositions of the Nb_5Si_3 in the bulk of the oxidised alloys at 800 °C and 1200 °C. The average concentrations of elements are in at.%.

Alloy	Nb	Ti	Si	Cr	Al	Sn	O	Si+Sn	Si+Sn+Al	Nb/Ti
800 °C										
ZX3	42.3	16.1	35.8	1		0.7	2.2	36.5		2.6
ZX5	44.7	15	35		1.8	0.6	3	36.1	37.4	3
ZX7	43.8	16.8	29.6	1.1	3.7	1.1	3.9	30.7	34.4	2.6
1200 °C										
ZX3	43.8	16.6	34.3	0.9		0.7	3.7	35		2.6
ZX5	44.2	14.9	34.6		1.7	0.6	4	35.2	36.9	3
ZX7	41.9	18	33.1	0.3	1.4	0.4	4.9	33.5	34.9	2.3

Table S17. Comparison of the compositions of the A15-Nb_3X in the bulk of the alloys ZX5 and ZX7 at 1200 °C. The average concentrations of elements are in at.%.

Alloy	Nb	Ti	Si	Cr	Al	Sn	O	Si/Sn	Si + Sn	Si+Sn+Al	Nb/Ti
ZX5	56.8	22	3.1		7.2	5.1	5.8	0.61	8.2	15.4	2.6
ZX7	51.5	18.1	2.4	4.7	9.6	6.9	6.8	0.35	9.3	18.9	2.8

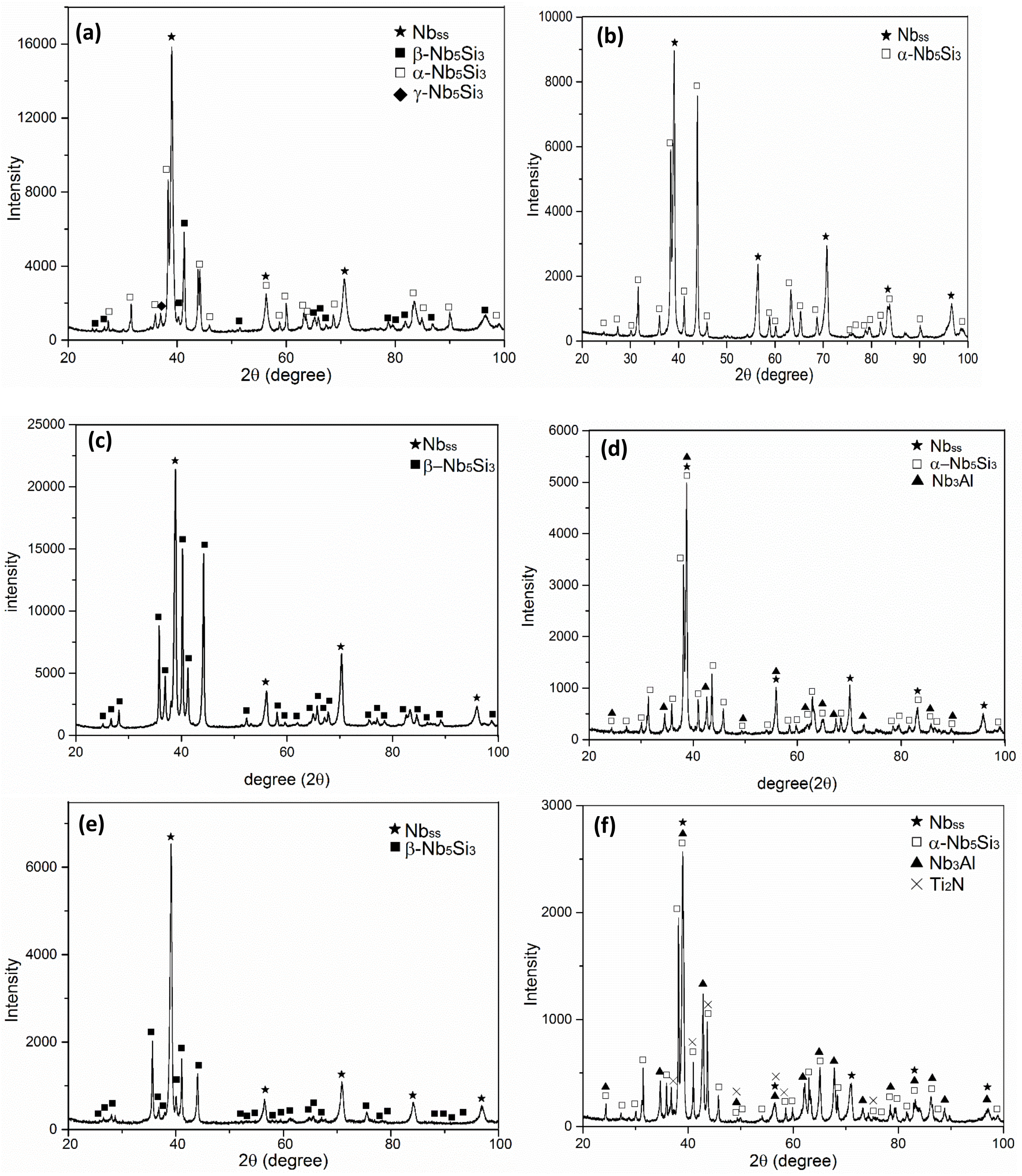

Figure S1 X-ray diffractograms of the cast and heat treated alloys (a) and (b) ZX3, (c) and (d) ZX5, (e) and (f) ZX7.

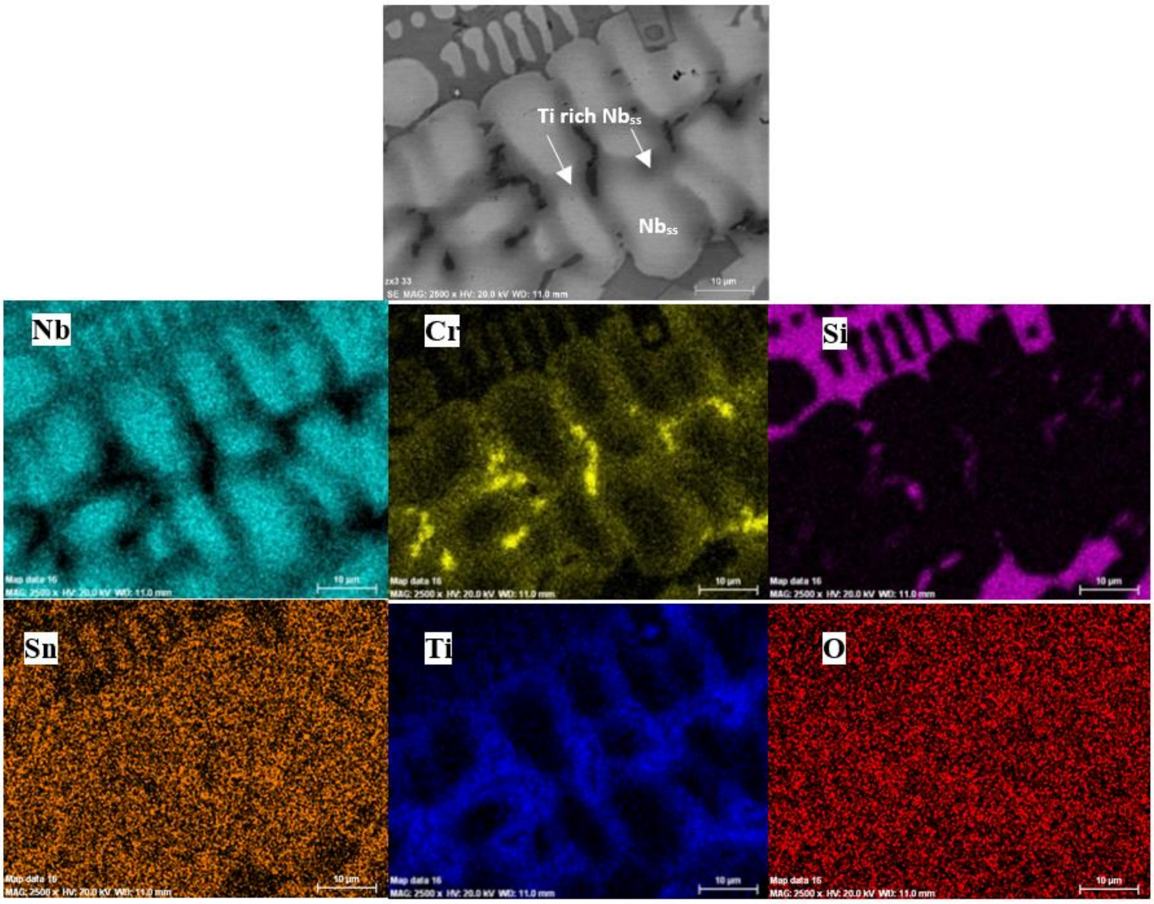

Figure S2 BSE image and X-ray maps showing the presence of a Cr rich phase (assumed to be the C14-$NbCr_2$ Laves phase) in the bottom of the button of the cast alloy ZX3.

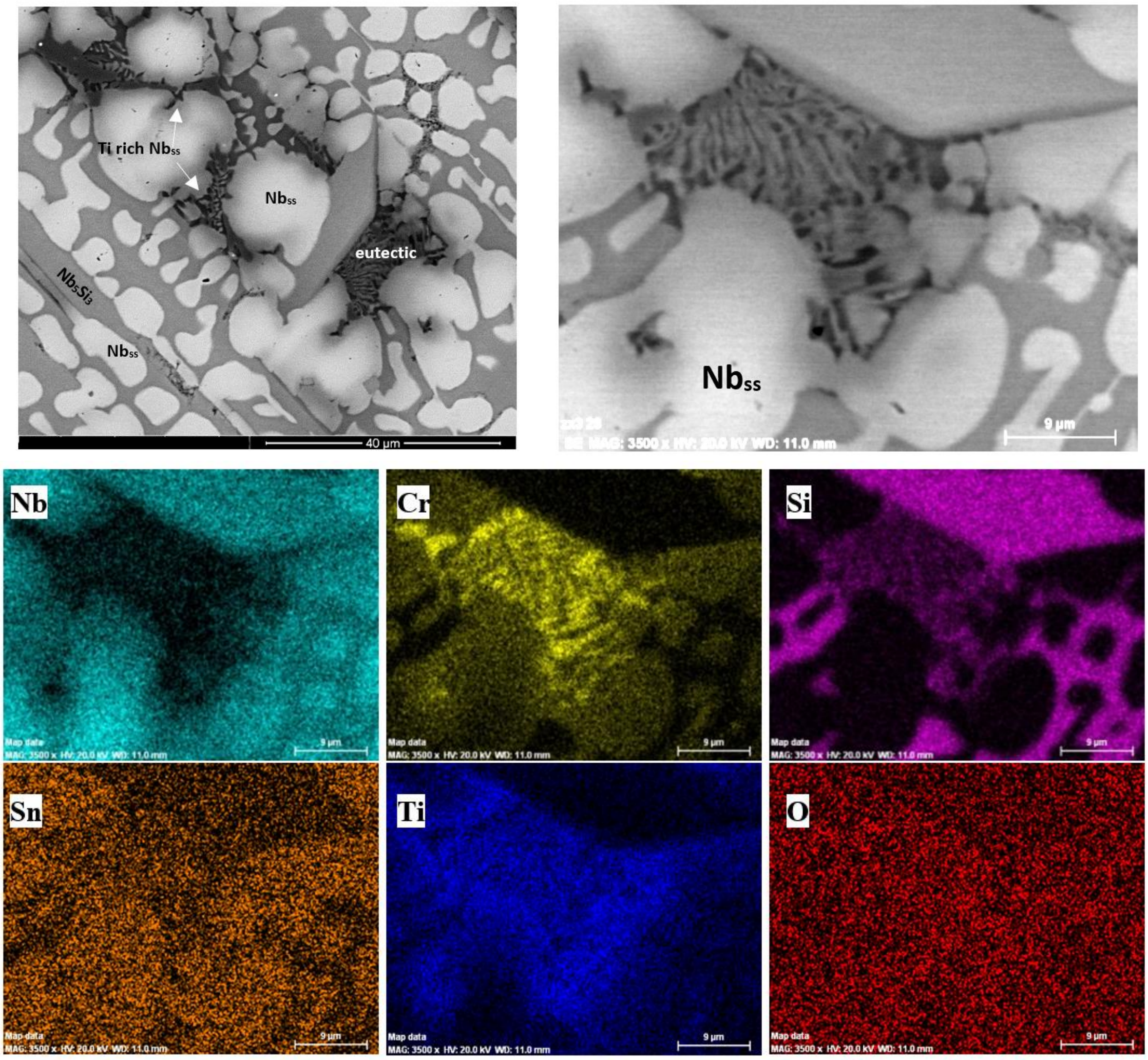

Figure S3 Top left, BSE image of the microstructure in the bottom area of the button of the cast alloy ZX3 showing areas with eutectic. Top right, BSE image of eutectic observed in the bottom of the button. X-ray elemental maps for the top right image.

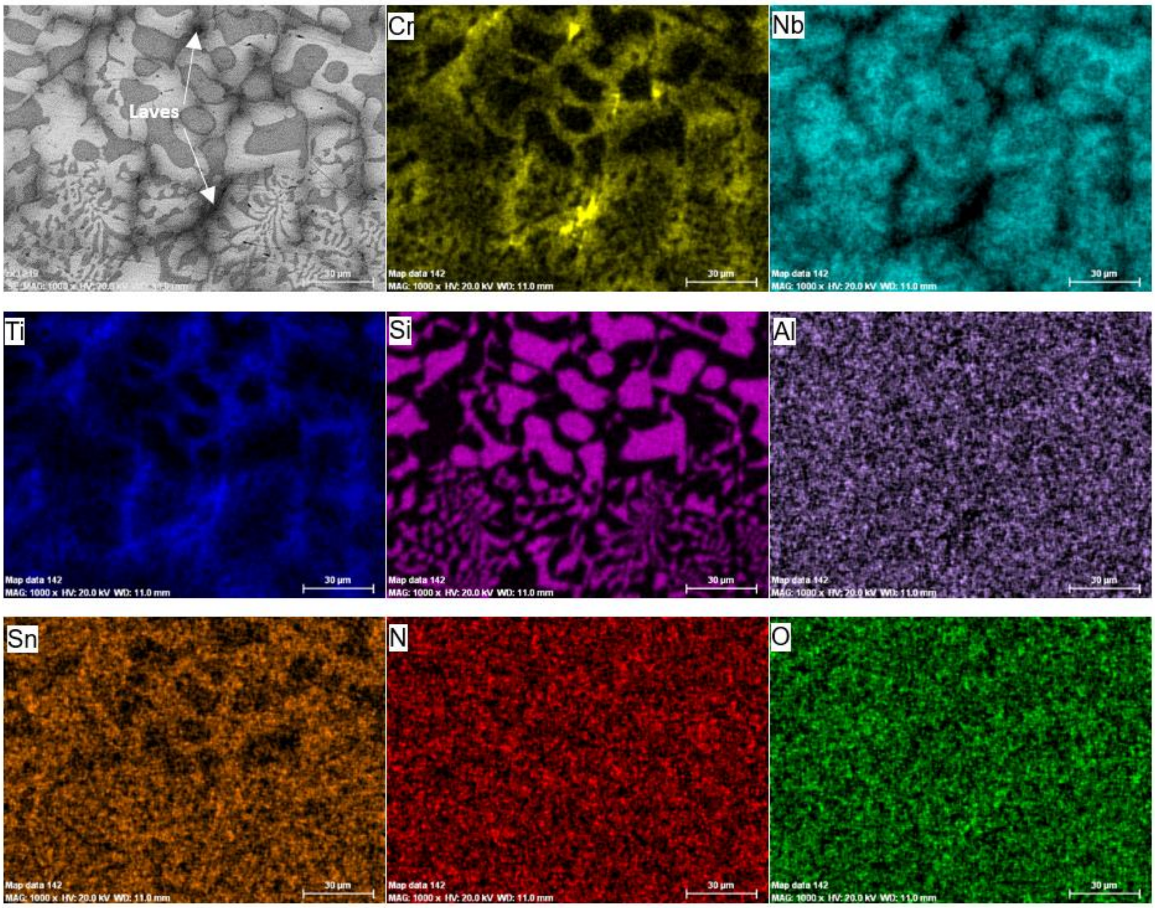

Figure S4 BSE image and X-ray elemental maps of the bottom area of the cast alloy ZX7.

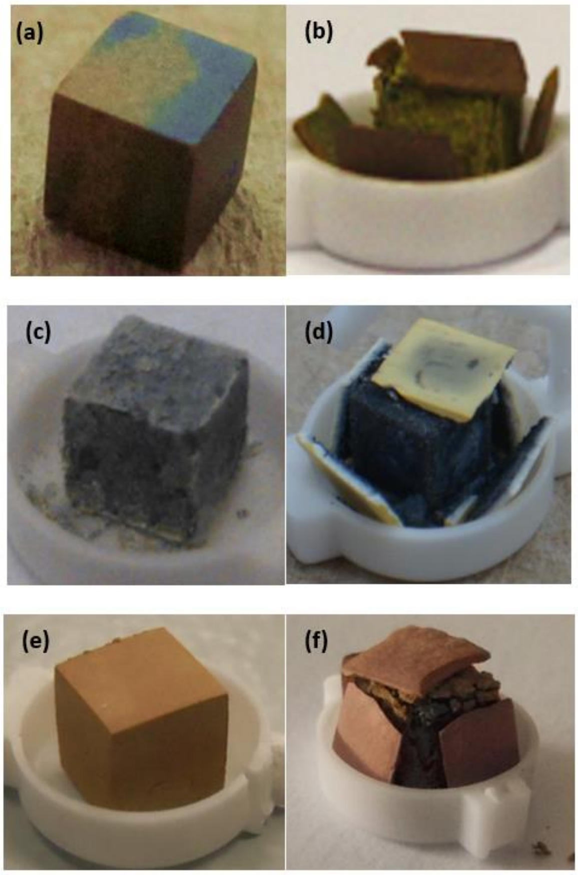

Figure S5 The oxidised specimens at 800 ^{o}C (a), (c) and (e) and at 1200 ^{o}C (b), (d) and (f). Images correspond to alloys as follows: (a) and (b) ZX3, (c) and (d) ZX5 and (e) and (f) ZX7.

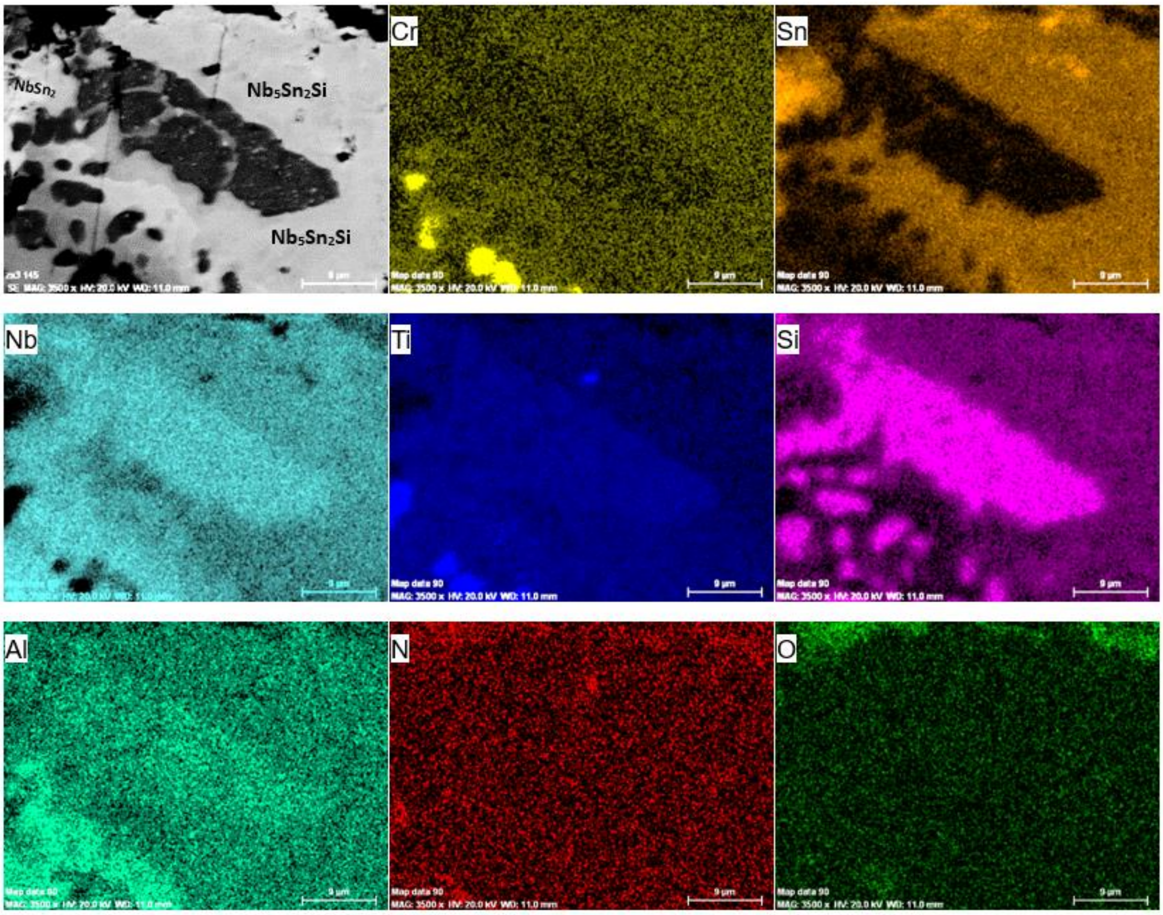

Figure S6 BSE image and X-ray elemental maps of the Sn rich zone of the alloy ZX7 after oxidation at 1200°C.
